# Study of the spatial distribution of vertical and longitudinal acceleration and sensor installation position of ballast track based on wheel-rail coupling model

**DOI:** 10.1371/journal.pone.0319803

**Published:** 2025-03-24

**Authors:** Ke Wang, Kun Zheng, Youjie Cai

**Affiliations:** 1 College of Mechanical and Electrical Engineering, Qiqihar University, Qiqihar, China; 2 The Collaborative Innovation Center for Intelligent Manufacturing Equipment Industrialization, Qiqihar University, Qiqihar, China; 3 College of Culture of Literature and History, Qiqihar University, Qiqihar, China; Tongji University, CHINA

## Abstract

In order to study the basic parameters and sensitive areas of track vibration acceleration during train passage, a vehicle-rail dynamic finite element model based on wheel-rail coupled dynamics is established. The vertical and longitudinal distributions of track vibration acceleration under load are calculated with the dynamic model at the vehicle speed of 250Km/h. And the experimental modal analysis is carried out using the ballastless track mechanical test platform with pulse hammer excitation. The study found significant spatial distribution characteristics of vertical and longitudinal accelerations at different positions along the rail; the root-mean-square value is more suitable than the maximum value to represent the vibration of the whole rail span, while the entropy value can be used to analyse the vibration of the rail; wheel-rail accelerometers are very sensitive to vibration energy outside the rail head, but installing accelerometers on the outside of the rail head is difficult and may affect travel safety; it is more appropriate to choose the waist part of the rail for detection.

## Introduction

**Fig 1:**
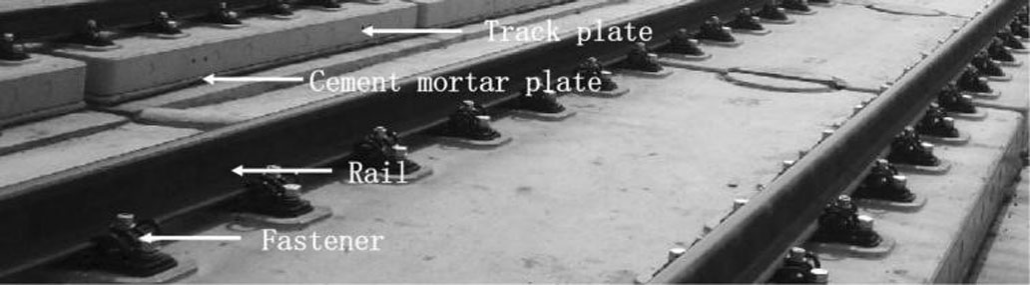
Continuous Welded Rail in Ballastless track.

In recent years, more and more attention has been paid to ensuring that high-speed trains operate safely. Based on the theory that the vibration characteristics of vibrating structures can be easily modified by their physical properties [1], there has been an increasing number of studies using vibration response for health monitoring of railway operations [2–10]. Ballastless track structures, which support the operation of high-speed railways, are also a clear focus of health monitoring studies. The structural components of the ballastless track structure include the rails, fasteners, track plates, mortar panels and foundations (Fig 1). The rail is an essential component of the track structure and its main function is to guide the vehicle forward and to support and transmit the wheel load [11]. Wheel-rail forces resulting from wheel-rail interactions act directly on the rails, generating vibration intensities that are more pronounced in the track structure [12,13]. Many researchers have contributed to the analysis of the physical properties of rail structures using rail vibration signals.

Zhu *et al.* [14] used the rail frequency response function to analyse the changes in the dynamic properties of the rail structure when the fastener support fails. Shi *et al*. [15] found that higher-order spectral analysis of the rail vibration response can be effectively used to detect and identify wheel flat scars. Song *et al*. [16] investigated the relationship between rail acceleration and corrugation wave depth. Bracciali and Cascini [17] monitored rail acceleration signals and processed them using synthetic energy and cepstrum analysis criteria to identify wheel-track defects. Maclean *et al*. [18] developed an intelligent biaxial strain sensor based on a micro-electro-mechanical system to detect the dynamic responses of specific wheels, train speed and track conditions at the rail waist. Wang *et al*. [19] established a three-dimensional model of wheel-rail coupled rolling contact for high-speed railways and found that the resonance phenomenon of the wheel-rail system at the dominant eigenfrequency of the vertical vibration acceleration of the rails and wheels led to an increase in the dynamic response of the wheel-rail system through field measurements. Zhan *et al*. [20] installed MEMS sensors in the rail waist to detect loose fasteners. Cai *et al*. [21] combined a high-speed rail vehicle model with a flat track model to establish a coupled vehicle-rail dynamics model, and conducted field tests on the Ha’erbin-Dalian (HD) high-speed railway line, and analysed the wheel-rail contact force by numerical simulation and experimental results using the rail acceleration.

In summary, many results have been achieved in the analysis and application of rail vibration signals and the measurement of railway operation health check. However, the rail as a three-dimensional solid structure, wheel-rail force excitation, its different regions of vibration response is obviously different. Effective monitoring for different inspection purposes requires the installation of sensors in different regions of the rail. Therefore, to solve this problem, it is necessary to study the spatial distribution characteristics of the rail, to explore the basic parameters of the rail vibration acceleration and the sensitive area in order to install the rail sensor more effectively. Cai *et al*. [22] from Southwest Jiaotong University established a rail dynamics model and a rail bottom finite element model based on GENSYS and ABAQUS, and investigated the basic parameters and sensitive areas of rail vibration during train passage. However, the research content of literature 26 is aimed at the vertical vibration acceleration of vertically distributed rails, and there is no research on the three-dimensional acceleration distribution in the vertical direction of the rails and the three-dimensional acceleration distribution along the longitudinal direction of the rails.

In this paper, a finite element simulation model of coupled rail dynamics is established and experimentally verified to study the vibration acceleration distribution of rails more systematically, and the vertical and longitudinal spatial distributions of three-dimensional rail acceleration distributions under train loads are analysed.

## Calculation model

### Wheel-rail coupling relationship

**Fig 2:**
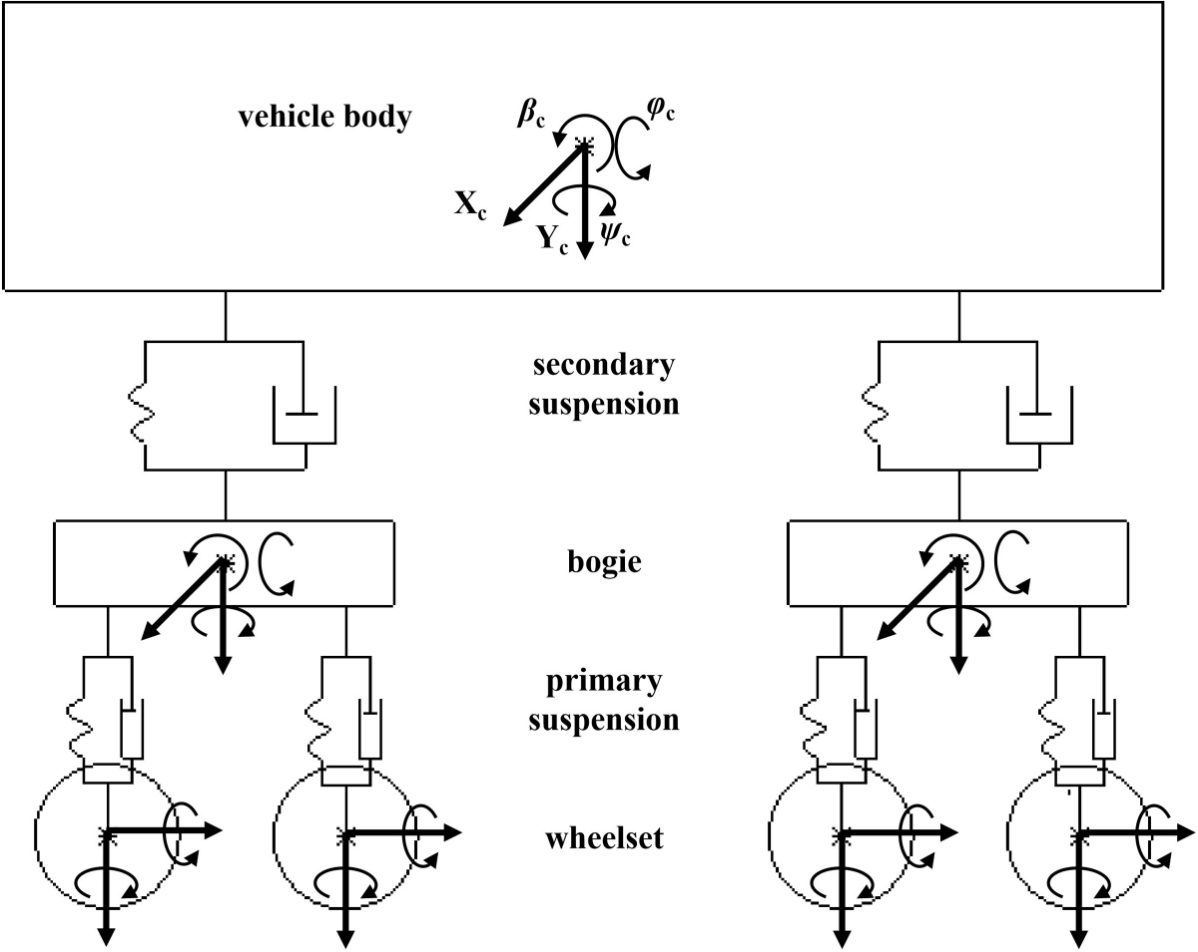
Vehicle model.

As shown in Fig 2, The vehicle track coupling dynamic model established in this study is mainly composed of a vehicle body, a bogie, a wheelset and a suspension system. The vehicle body, bogie, and wheelset form a multi-rigid body system of the vehicle model, which is connected by secondary suspension systems [23].

The wheel–rail coupling contact force includes the normal and tangential forces. The wheel tread is modeled according to the LM worn type of locomotive wheel [24].

The wheel-rail normal force plays a key role in the vehicle track coupling system, which can be determined by the Hertz nonlinear elastic contact theory is expressed as follows [25]:


$$p\left(t\right)= \{\begin{array}{c} {\left[\frac{1}{G}\delta Y\left(t\right)\right]}^{\frac{3}{2}}\delta Y\left(t\right)>0\\ 0\delta Y\left(t\right)\leq 0 \end{array}$$ (1)

Where

*δY(t)* elastic compression between the wheel and rail (*m*);

*G* wheel-rail contact constant (*m/N*^*3/2*^), which is related to the tread form.


$$\delta Y\left(t\right)=Y_{wj}\left(t\right)-Y_{r}\left(x_{wj},t\right)-Y_{irr}\left(t\right)$$ (2)

Where, *j = 1*, *2*, *3*, and *4* represent wheels *1*, *2*, *3*, and *4*, respectively; *Ywj* (*t*) and *Yr* (*xwj*, *t*) are the displacements of the *j-th* wheel and rail at the wheel-rail contact, respectively; *Yirr* (*t*) is the track irregularity value. Obviously, when *δY*(*t*) ≤ 0, the wheel and rail are separated from each other, do not contact each other, and the wheel-rail force is zero.


$$G= \{\begin{array}{c} 4.57R^{-0.149}\times 10^{-8}\left(\text{Conical tread wheel}\right)\\ 3.86R^{-0.115}\times 10^{-8}\left(\text{Wear shaped tread wheel}\right) \end{array}$$ (3)

Where, *R*(*m*) is the rolling radius of the wheel.

Rolling and sliding occur between wheels and rails. Since the relative sliding between wheel and rail is extremely small, the wheel-rail rolling contact theory, which studies this relative motion phenomenon, is called the wheel-rail creep theory. The coefficient of friction between wheel and rail gradually decreases and tends to flatten as the relative sliding rate increases [26]. It is necessary to use the exponential decay model to characterize the variation of the wheel-rail friction coefficient with the relative sliding rate, which is consistent with the variation law of the friction coefficient in the creep theory [27].

The contact tangential stress can be defined as the coulomb friction model [28,29].

According to theory of coulomb friction:


F=  pμ (4)

Where

*μ* friction coefficient;

*P* normal contact pressure between contact surfaces.

The conversion between static and dynamic friction coefficients in the exponential decay model is expressed as:


$$\mu =\mu k+\left(\mu s-\mu k\right)e-\beta v$$ (5)

Where

*μs* static friction coefficient;

*μk* dynamic friction coefficient;

*β* attenuation coefficient;

*v* sliding speed of main contact surface and secondary contact surface.

The wheel-rail tangential force is the sum of the frictional force in the wheel-rail contact sliding area and the viscous resistance in the wheel-rail contact adhesion area, which can be expressed as:


F=pμ+cv (6)

Where, *c* is the relative speed of wheel-rail nodes.

### Vehicle track dynamic model

In the dynamic simulation model, the vehicle model is simulated using Abaqus simulation software and the following assumptions are made:

The car body, bogie and wheel set are rigid bodies with no elastic deformation. The longitudinal vertical plane through the center of mass is left-right symmetric, and the transverse vertical plane is front-back symmetric;

As shown in Fig 2, the vehicle suspension system obeys Hooke’s law. The damping is viscous damping, and the body and bogie have five degrees of freedom: roll, nod, sway, vertical, and horizontal. The wheelset has four degrees of freedom: roll, sway, vertical, and horizontal. The vehicle has seven rigid bodies with 31 degrees of freedom.

**Fig 3:**
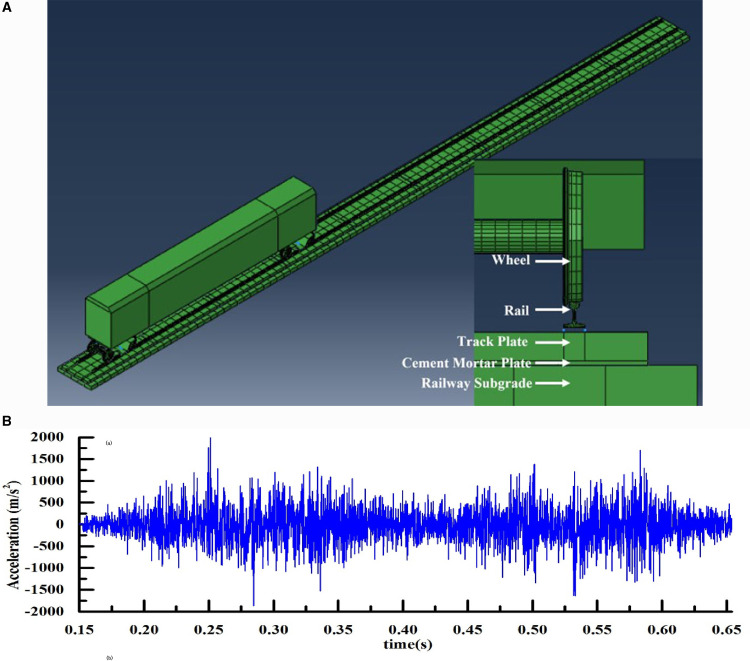
a. Simulation model of track-train coupling system. b. Simulation result.

As shown in Fig 3a, the structures under the wheel include the rail, fasteners, ballastless track plate, cement mortar, and foundation. The fasteners are simulated using spring damping elements, and the others are simulated using deformable solid elements. The track model is set to a continuous elastic support mode. The rail and the track plate are connected by fasteners, which are simulated by springs and viscous dampers.

As shown in Fig 3a, the steel rails, track plates, mortar plates and foundation are fixed at both ends. The vehicle length is 25m, the vehicle spacing is 18m and the bogie wheelbase is 25m. The nominal rolling circle diameter of the wheels is 860mm, the body mass is 45480 kg, the bogie mass is 3300 kg and the wheelset mass is 1780 kg. The vehicle runs at a speed of 250km/h and the material parameters are listed in Table 1. The simulation results obtain the X-axis acceleration in the outward direction of the vertical rail waist, the Y-axis acceleration in the downward direction of the vertical rail head, and the Z-axis acceleration along the rail direction.

**Table 1:** Components parameters of the model.

Rail	Type(kg/m)	60
	Length(m)	20
	Cross-sectional area (mm^2^)	7745
	Elastic modulus (GPa)	210
	Poisson ratio	0.3
	Coefficient of linear expansion (°C·mm/m)	0.0118
WJ-7 Fasteners	Distance between fasteners (mm)	630
	Thickness of under-rail pads (mm)	10
CRTS-1 Track plate	Elastic modulus (GPa)	36
	Poisson ratio	0.2
	Density (kg/mm^3^)	2.5e-6
	Coefficient of linear expansion (°C·mm/m)	0.01
Cement mortars	Thickness (mm)	50
	Elastic modulus (GPa)	0.3
	Density (kg/mm^3^)	1.8e-6
	Coefficient of linear expansion (°C·mm/m)	0.01
	Friction coefficient of contact surface on track plate	0.35
	Friction coefficient of contact surface on subgrade	0.35
Railway Subgrade	Thickness (mm)	300
	Elastic modulus (GPa)	33
	Density (kg/mm^3^)	2.5e-6
	Coefficient of linear expansion (°C·mm/m)	0.01

As shown in Table 1, the technical parameters used in the vehicle model, which refer to CRH2 [30] electric multiple units, are listed. The rail uses a solid finite element model of 60 kg/m section based on standard dimensions [31]. The finite element model of the rail system is shown in Fig 3a.

As shown in Fig 3b, the results of 250 km/h rail acceleration and are calculated by the model. The model simulation results show acceleration values in the range of [-1872.12 m/s2, 2159.87 m/s2]. The field measured acceleration range of high-speed railway line is 1000 ~ 3000 m/s2, and the acceleration is in the acceptable range. The waveforms and peaks of the calculated results in this paper are all in the order of magnitude compared with the range of the test results. The indicators are all within the range of the test results, and the calculated results of the dynamic simulation in this paper are reasonable.

### Distribution of rail inspection points

**Fig 4:**
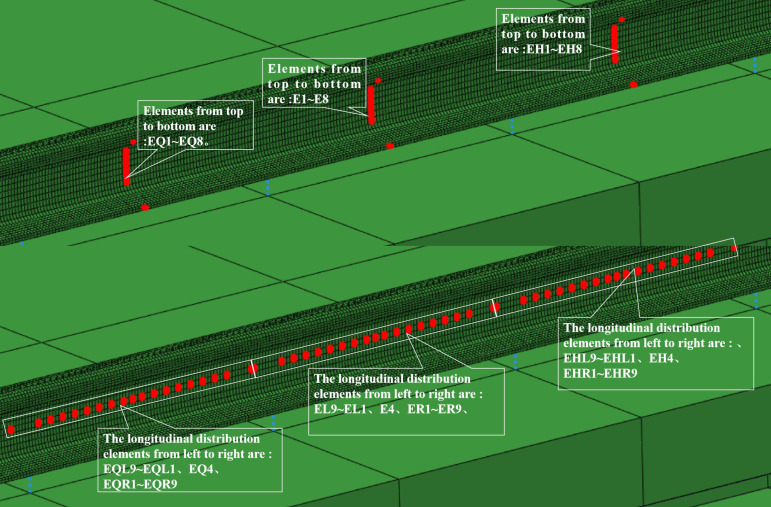
Location of data acquisition points of simulation model.

Considering the difficulty and acceptability of field engineering inspection, vertical and longitudinal series inspection elements are selected outside the rail, as shown in Fig 4. In the middle of the rail span, three series of detection elements distributed from the top side to the bottom side of the rail are set: EH, E and EQ series elements. The rail neutral axis positioning elements are EQ4, E4 and EH4. On the neutral axis of the rail, several series of detection elements have been set longitudinally along the rail and distributed in three consecutive spans of the rail: EQL, EQR, EL, ER, EHL, EHR series elements.

## Simulation and experimental analysis

### Vibration acceleration analysis of vertical distribution detection points

Referring to Fig 4, based on the calculation results of E-series, EQ-series and EH-series elements of the vertical distribution elements, the accelerations of these elements are analyzed. The serial numbers 1-8 indicate the location of the serial number of the corresponding elements. X_E_, X_EQ_ and X_EH_ are the transverse accelerations of each element in the three vertical distribution series. Similarly, Y_E_, Y_EQ_ and Y_EH_ are the vertical accelerations of each element, and Z_E_, Z_EQ_ and Z_EH_ are the longitudinal accelerations of each element.

**Fig 5:**
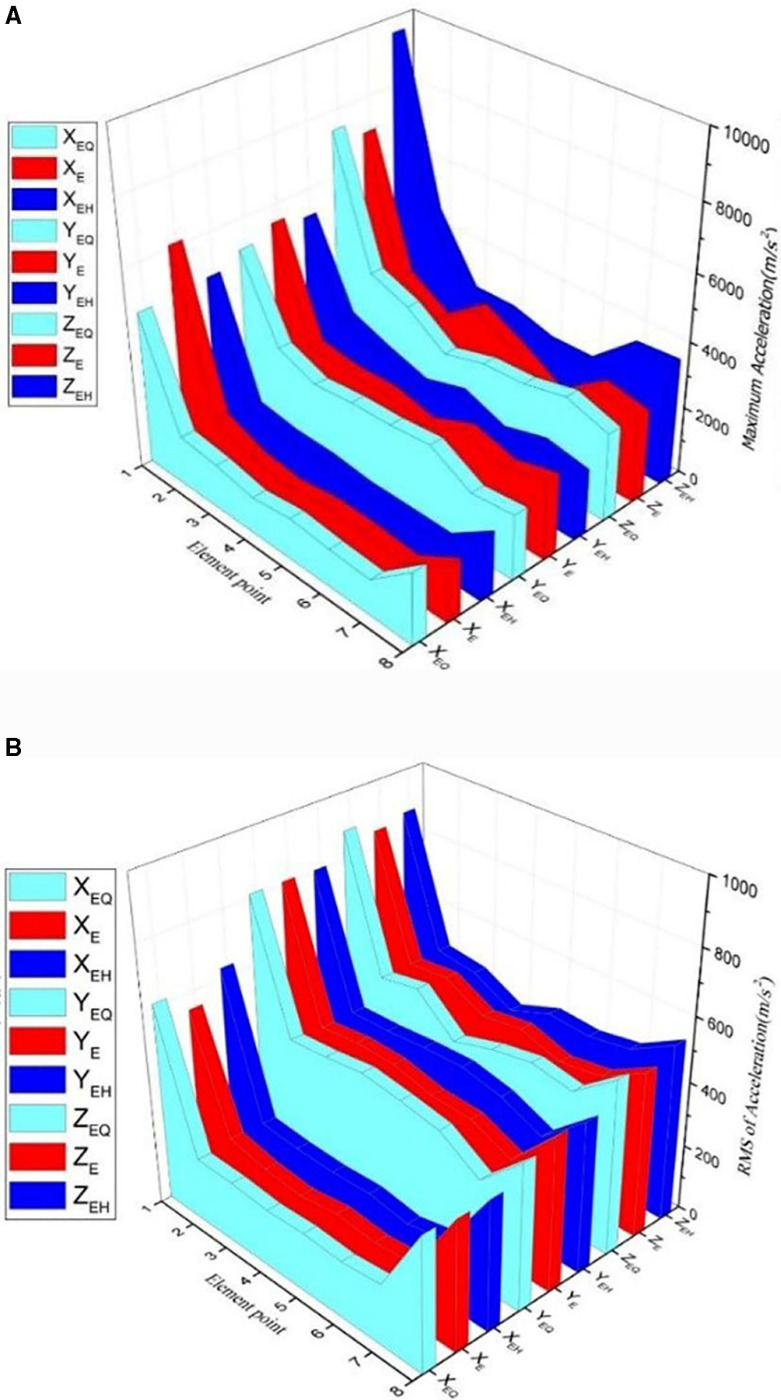
a. The maximum acceleration of vertical distribution element. b. The RMS of vertical distribution element.

As shown in Fig 5a, the maximum acceleration of the serial number of the vertical distribution element is calculated. The maximum acceleration of each vertical distribution element along the vertical direction is different. The maximum acceleration of the rail- head side elements E_1_, E_H1_, and E_Q1_ is the largest, which is almost twice that of the other positions, and the longitudinal acceleration value is the largest. The transverse acceleration in the rail waist element is smaller than that in the other directions.

As shown in Fig 5b, the root mean square (RMS) of each element is calculated for the element series. The RMS values of the three vertically distributed accelerations are different in the three directions. The gap between the rail head and rail bottom is large, and its value at the rail waist is relatively close. The value at the rail head is the largest, followed by the rail bottom, and then the rail waist.

Based on the analysis of Fig 5, the vertical distribution of rail vibration has similar characteristics at different locations for the same vehicle speed. As shown in Fig 5a, peak changes can be caused by transient events which only reflect the maximum intensity of the vibration signal at a particular time and cannot represent the characteristics of the entire vibration process, resulting in poor stability and repeatability. As shown in Fig 5b, the RMS value provides a measure of the average power level and is less sensitive to outliers, reducing the effect of these transient events on the results. This makes the RMS value more stable and reproducible under different test conditions, and more comprehensive in reflecting the characteristics of vibration signals. Therefore, using RMS values instead of peak acceleration makes it easier to investigate the distribution of rail acceleration. The vibration of each part of the rail is significantly different during the train operation, and the solid simulation is more suitable to study the rail vibration.

### Vibration acceleration analysis of longitudinal distribution detection points

Referring to Fig 4, based on the calculation results of the longitudinally distributed elements, the accelerations of these elements in the three directions are analyzed. Horizontal coordinates from left to right for the position sequence number of the longitudinally distributed elements. X_E_, X_EQ_ and X_EH_ are the transverse accelerations of each element in the three longitudinal distribution series. Accordingly, Y_E_, Y_EQ_ and Y_EH_ are the vertical accelerations of each element, and Z_E_, Z_EQ_ and Z_EH_ are the longitudinal accelerations of each element.

**Fig 6:**
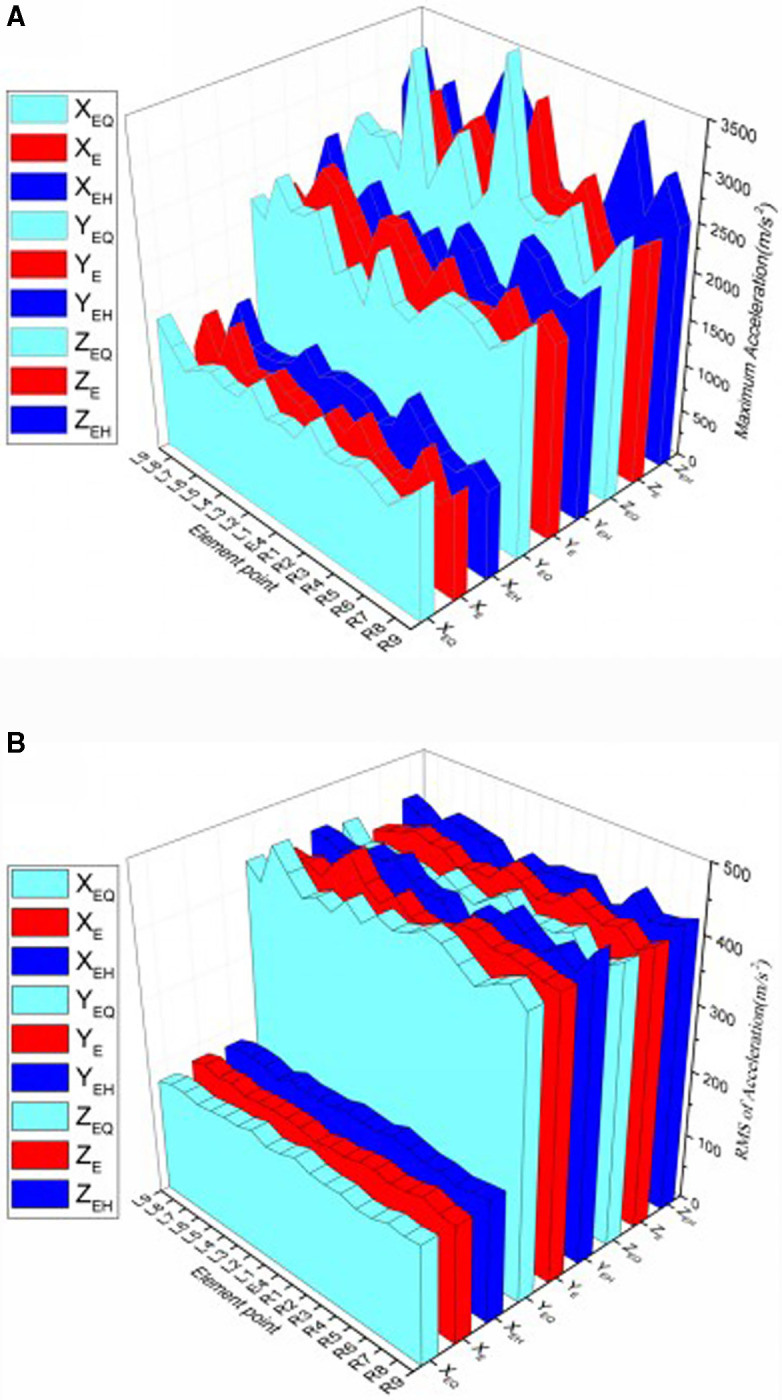
a. The maximum acceleration of longitudinal distribution element. b. The RMS of longitudinal distribution element.

As shown in Fig 6a, the maximum acceleration of each element in the longitudinal distribution of the different rail spans is calculated. The maximum acceleration value of the longitudinal element position in different rail spans also varied. The maximum value of transverse acceleration is much smaller than vertical and longitudinal acceleration.

As shown in Fig 6b, the RMS values of each element in the longitudinal distribution of the different spans of the rail are calculated. The fluctuation in the peak value is smaller and more stable than the maximum value. The RMS values of vertical acceleration and longitudinal acceleration are similar and more than twice those of the transverse acceleration. The RMS values of the same element position along the longitudinal distribution of the different rail spans are different, and the acceleration of the same element along the longitudinal distribution of the same span is also different.

**Fig 7:**
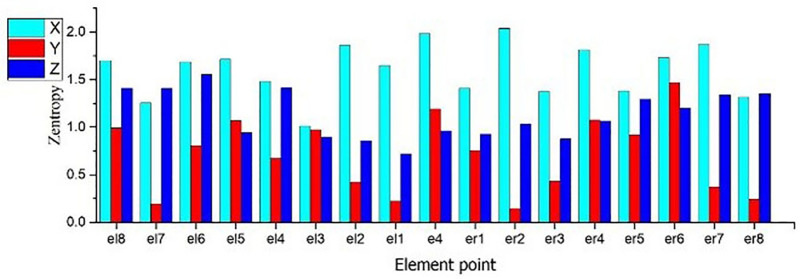
Entropy of Longitudinal distribution element.

As shown in Fig 7, the information entropy of the E-series elements in the longitudinal distribution is calculated. The information entropy of the acceleration in the three directions fluctuates along the longitudinal direction when the rail is excited. The entropy of the vertical acceleration in the three directions are generally low, and fluctuated along the longitudinal direction, and it is high at the middle rail waist.

## Experiment and research on rail vibration characteristics

### Experimental device

In this section, the distribution of rail acceleration is investigated by using the ballastless track rail mechanical test platform [32]. The experimental platform mainly includes rail, end fixing device. In the paper we choose CRTSІ slab Ballastless Track to establish the experimental system. The rail is 60 kg/m and is locked with WJ-7 rail fasteners with a preload torque of 100NM. Rail fastening space is 600mm. End fixing device connect the rail with reinforced concrete foundation by screw bolt.

**Fig 8:**
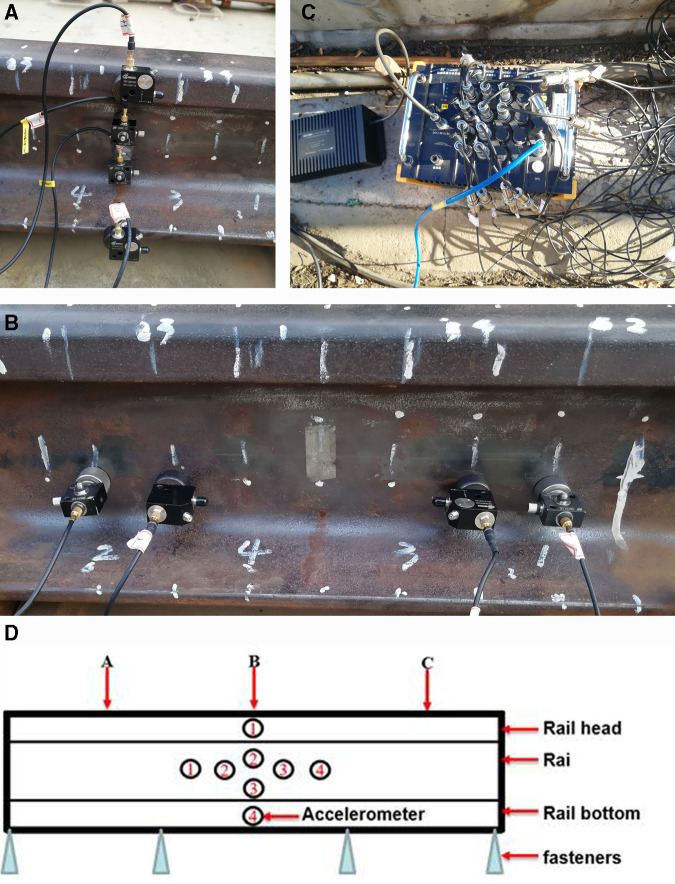
a. Vertically distributed acceleration sensors. b. Longitudinal distributed acceleration sensor. c. Test instrument. d. Structure diagram of distribution acceleration.

The vertical (Fig 8a) and longitudinal (Fig 8b) distribution of rail vibration acceleration are studied, respectively. As shown in Fig 8c, the test experiment is DH5902 Data acquisition and analysis system (Donghua Testing Technology Co., Ltd).

As shown in Fig 8d, in the vertical direction of the rail center, one sensor is placed on the side of the rail head (position 1), two sensors are placed on the rail waist (positions 2 and 3), and one sensor is placed on the rail bottom (position 4). Four more sensors are placed along the longitudinal direction of the rail at the neutral axis position, located at 100 mm and 200 mm on either side of the rail center. A rubber hammer is used to excite position A on the left rail, position B on the middle rail, and position C on the right rail. The sampling frequency is 5000 Hz.

### Vibration acceleration analysis of vertical distribution detection points

**Fig 9:**
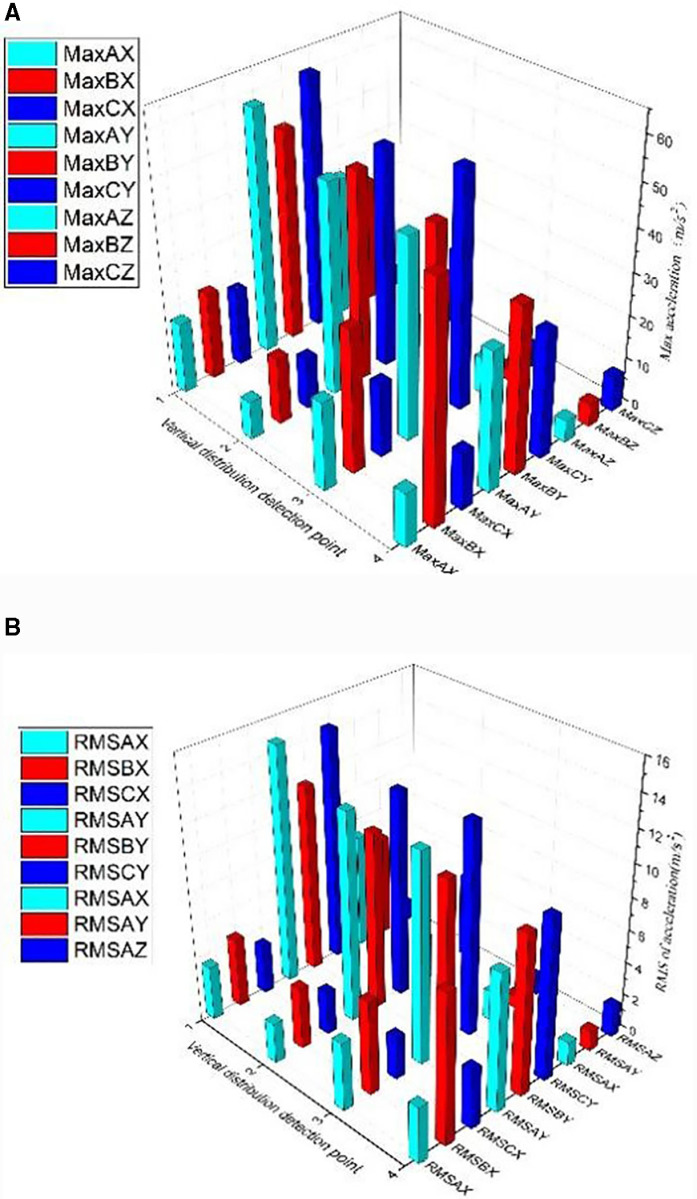
a. The Maximum acceleration of vertical distribution acceleration. b. The RMS of vertical distribution acceleration.

As shown in Fig 9, the maximum (Fig 9a) and RMS (Fig 9b) values of the vertical acceleration distribution in the vertical hammering experiment are analyzed and calculated. As hammering cannot control the magnitude of the excitation force, the three excitation positions are not compared horizontally. Comparing the simulation results of Fig 5, the acceleration at each position in the vertical distribution can be obtained.

The maximum acceleration and RMS values in different directions at different positions are different. The closer the excitation point, the higher the experimental value. Under the same excitation, the transverse and longitudinal acceleration responses are small, while the vertical acceleration response is large. The maximum and RMS values of transverse acceleration at the rail head and bottom are larger than those at the rail waist. The maximum and RMS value of the vertical acceleration at the rail head is the highest, while the value at the rail waist is close, and the difference between the rail head and bottom is large. The difference between the maximum value and the RMS value of the longitudinal acceleration at the rail head is highest, while the difference at the rail waist is close.

**Fig 10:**
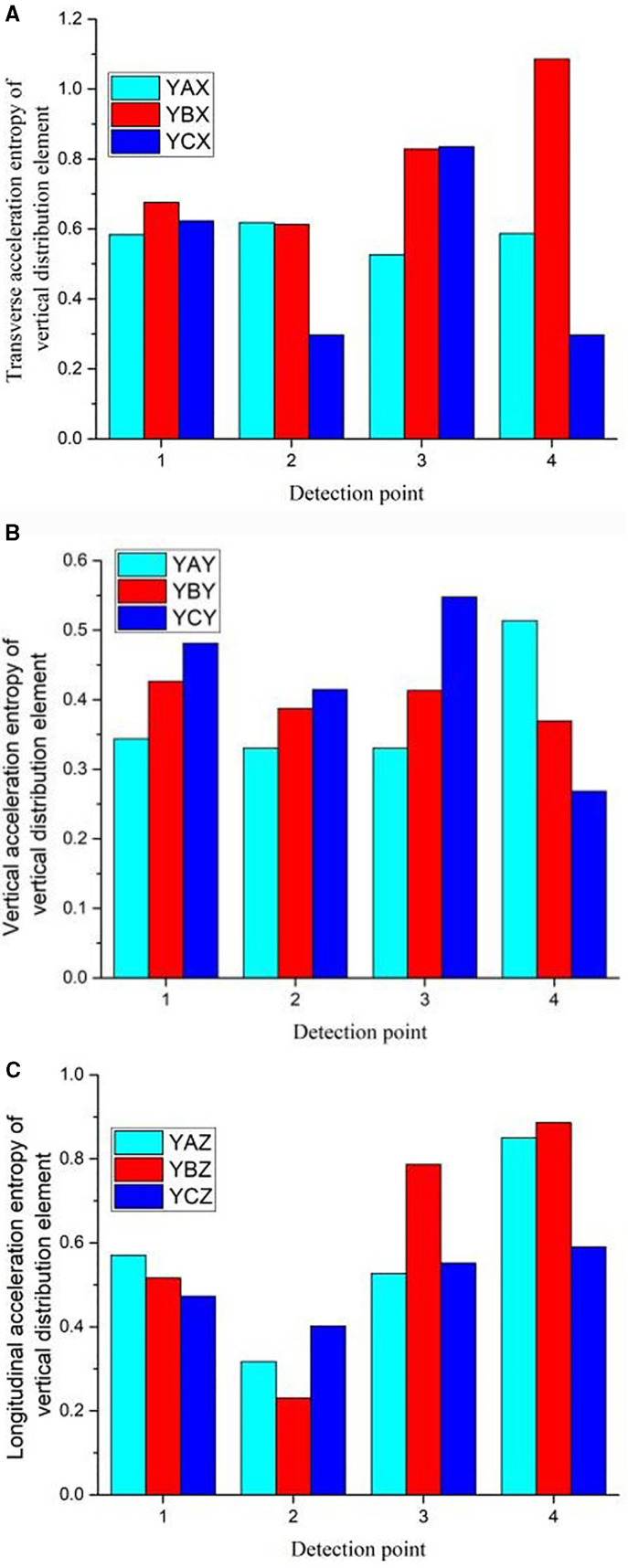
a. Entropy of vertically distributed transverse acceleration. b. Entropy of vertically distributed vertical acceleration. c. Entropy of vertically distributed longitudinal acceleration.

**Fig 11:**
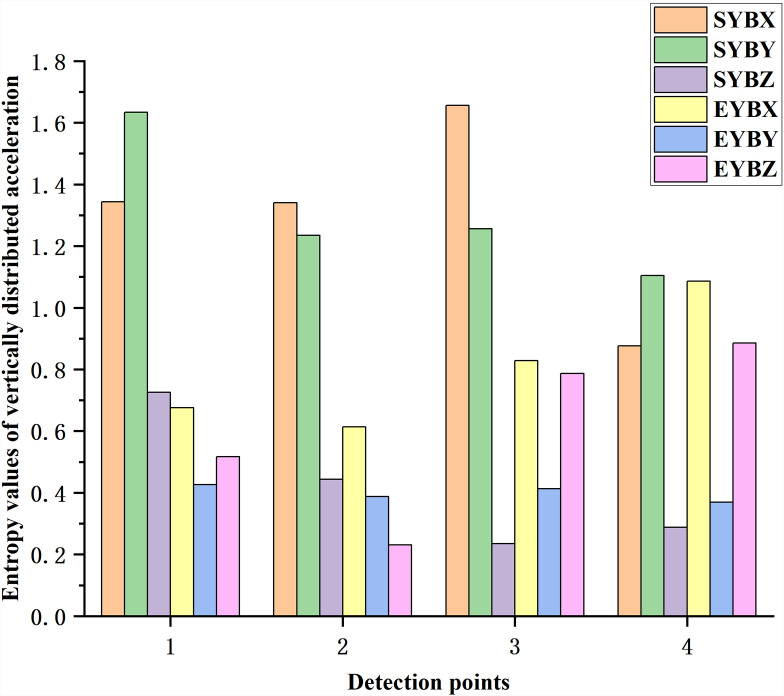
Comparison of entropy values of vertically distributed acceleration detection points between experimental and simulation results of hammering at position B.

As shown in Fig 10, in terms of information entropy, hammering at different positions affected the variation law of the same series, and the distribution of entropy values is similar. As shown in Fig 11, the entropy values of the simulation results of simulation units e1, e3, e6 and e8, which are similar to the detected sensor positions in the hammering experiment at position B, are selected for comparison. In the figure, SYBX, SYBY, SYBZ, EYBX, EYBY and EYBZ represent the simulated vertical distribution transverse acceleration entropy, the simulated vertical distribution longitudinal acceleration entropy, the simulated vertical distribution longitudinal acceleration entropy, the experimental vertical distribution transverse acceleration entropy, the experimental vertical distribution longitudinal acceleration entropy and the experimental vertical distribution longitudinal acceleration entropy, respectively. Where the first letters S and E denote the simulation and experimental results respectively, the second letters X, Y and Z denote the transverse, vertical and longitudinal distributions respectively, the third letter denotes the hammering position and the fourth letters X, Y and Z denote the transverse, vertical and longitudinal accelerations respectively, similarly below. The entropy value of the transverse acceleration (Fig 10a) is higher at the rail waist. The vertical acceleration (Fig 10b) is progressively smaller from the rail head to the rail bottom and the difference in excitation energy makes the measured entropy value smaller than the simulated value. The total variation of longitudinal acceleration is smaller in the longitudinal distribution. The change in magnitude of the entropy values at the four longitudinal acceleration (Fig 10c) measurement points is not significant.

**Fig 12:**
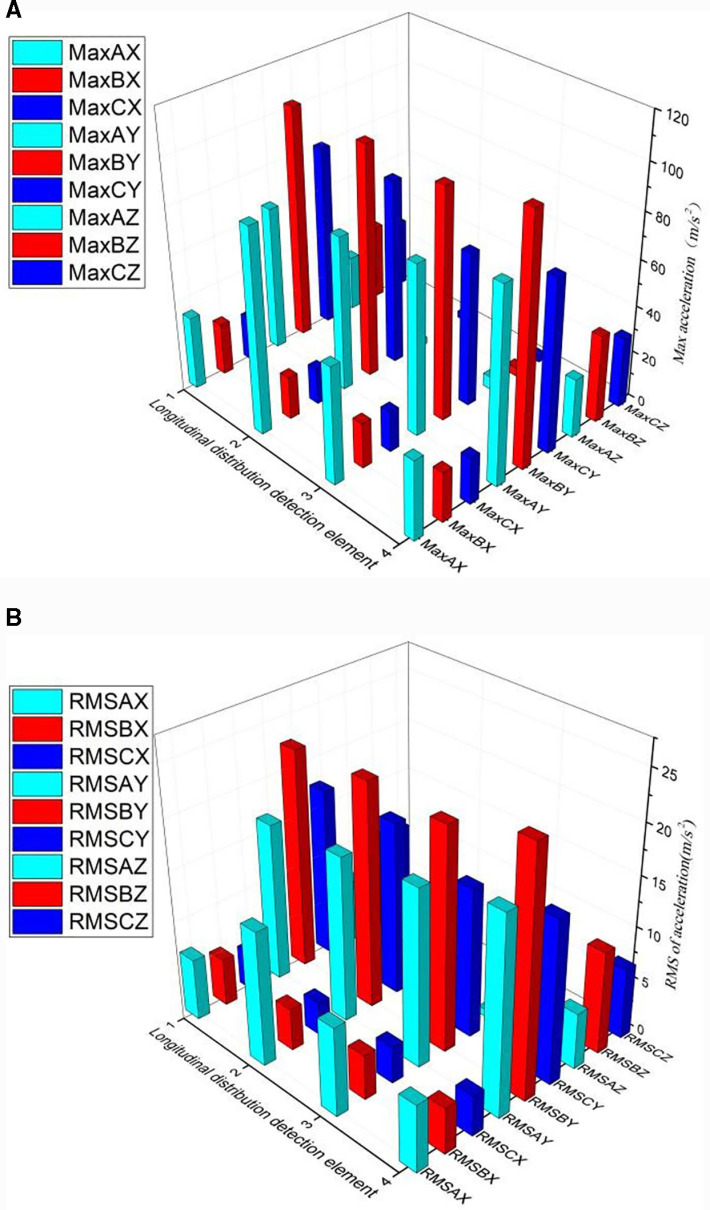
a. The Maximum acceleration of longitudinal distribution acceleration. b. The RMS of longitudinal distribution acceleration.

As shown in Fig 12, the longitudinal acceleration distribution is analyzed. As shown in Fig 12a, the closer the distance between the excitation point and the response point, the higher the response energy. The transverse acceleration response is the lowest and the vertical acceleration response is the highest for the same excitation. When hammering at the same position (e.g., position B), the longitudinal distribution of the RMS value (Fig 12b) of the acceleration showed an inconspicuous fluctuation. Compared to Fig 6, the maximum acceleration of each element in the longitudinal distribution of the rail fluctuates significantly, but the RMS value is unremarkable.

**Fig 13:**
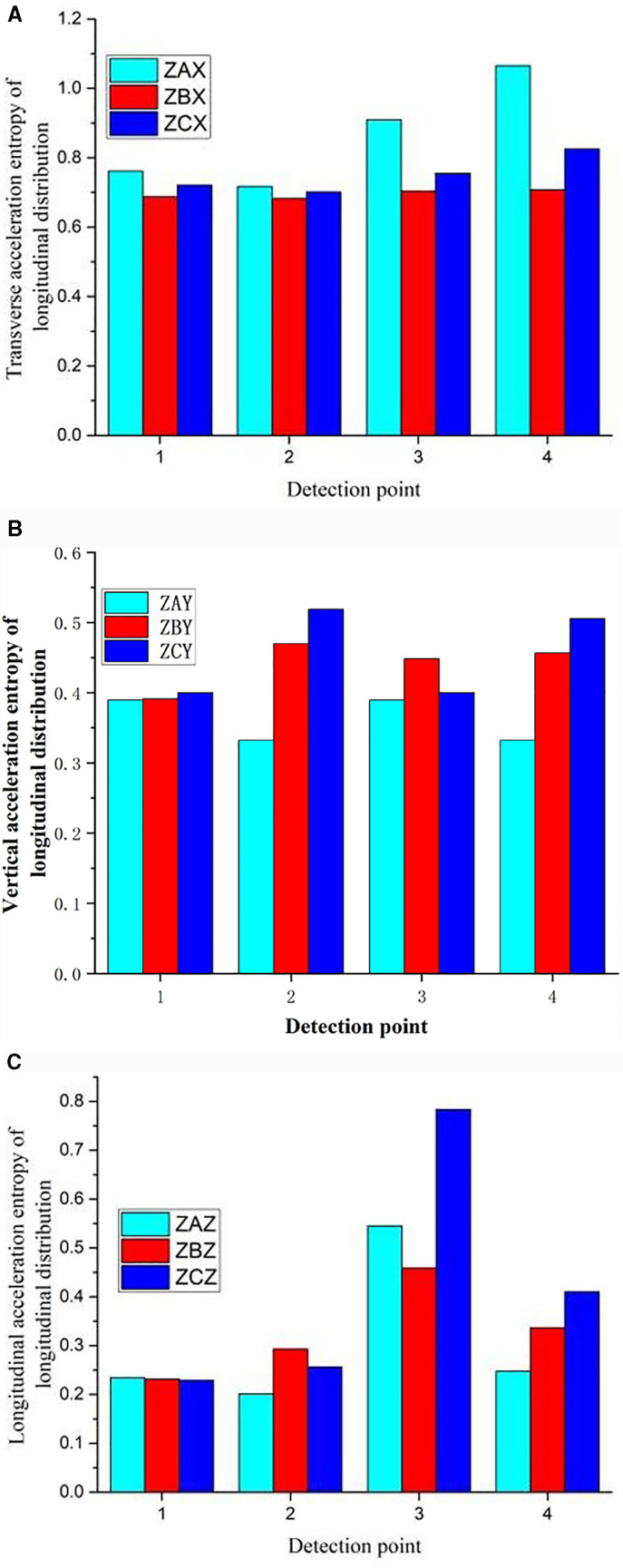
a. Entropy of longitudinal distribution transverse acceleration. b. Entropy of longitudinal distribution vertical acceleration. c. Entropy of longitudinal distribution longitudinal acceleration.

As shown in Fig 13, in terms of information entropy, hammering at different positions affects the variation law of the entropy values of the same series elements, and the distribution of entropy values is similar. The entropy of transverse acceleration is generally higher than that of vertical and longitudinal acceleration, and its value does not fluctuate in the longitudinal direction.

**Fig 14:**
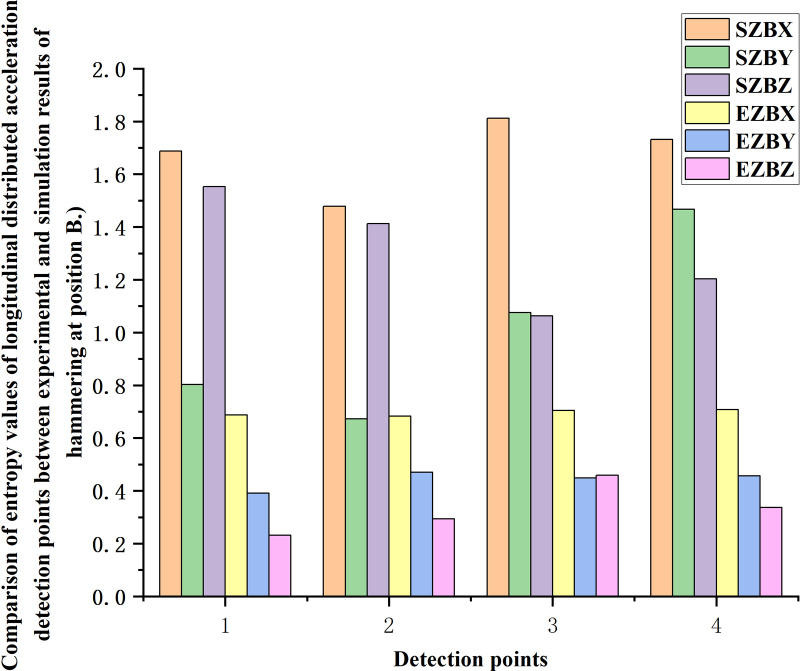
Comparison of entropy values of longitudinal distributed acceleration detection points between experimental and simulation results of hammering at position B.

As shown in Fig 14, the entropy values of the simulation results of simulation units el6, el4, er4 and er6, which are similar to the detected sensor positions in the hammering experiment at position B, are selected for comparison. The entropy value of the longitudinal distributed acceleration is relatively stable at the position near the rail, and the entropy value is low.

## Conclusion

1) The wheel-rail impact acts directly on the rail head, so the acceleration of the rail head in the vertical spatial distribution of the rail is significantly greater than the acceleration of the rail waist and the rail bottom. The elastic damping property of the fastening system has no significant effect on the acceleration at any position in the longitudinal spatial distribution of the rail, and the transverse acceleration is weaker in the three directions.
2) Although acceleration sensors are very sensitive to the vibration energy outside the rail head, the measurement results may be inaccurate due to factors such as impact and friction caused by the contact between the wheels and the track, and it is extremely difficult to install them outside the rail head, which may affect running safety. The vibration of the rail waist not only provides a more comprehensive understanding of the mechanical properties and operating conditions of the rail, but also the shape of the rail waist is relatively slender and smooth, making it easy to install acceleration sensors and maintain close contact with the rail surface, which can improve the accuracy and reliability of measurements. Therefore, installing acceleration sensors at the waist of the rail for detection is currently the preferred choice.
3) For the vertical distribution of acceleration, the maximum and effective values of transverse acceleration are greater at the rail head and rail base, and smaller at the rail waist. The vertical acceleration at the rail waist is relatively close, and there is a large difference between the rail head and rail base. The difference between the maximum and effective longitudinal acceleration values of the rail head and rail toe is closer than the other two directions. In terms of information entropy, the transverse acceleration spectrum has a larger entropy value at the waist of the rail, while the vertical acceleration spectrum has a larger entropy value at the head and toe of the rail. There is no obvious pattern in the amplitude variation of the entropy values between the four measurement points of the longitudinal acceleration.
4) For the longitudinal distribution of acceleration, there is not much difference between the maximum and effective values of acceleration in the transverse and vertical directions, but there are significant differences in the longitudinal acceleration response at different positions, and the vibration is slower near the centre of the track. The entropy value of the longitudinal distributed acceleration is relatively stable near the centre of the track, and the entropy value is small. Whether the rail vibration is analysed from the perspective of maximum value, effective value or entropy value, it shows that the vibration situation of each position of the rail is different. In order to effectively detect the steel rails, the vibration at the detection point must be relatively stable and reflect the overall vibration situation of the steel rotation. Therefore, the rail waist position is obviously a suitable detection point.

## Supporting information

S1 Fig Continuous Welded Rail in Ballastless track.
(TIF)

S2 Fig Vehicle model.
(TIF)

S3a Fig Simulation model of track-train coupling system.
(TIF)

S3b Fig Simulation result.
(TIF)

S4 Fig Location of data acquisition points of simulation model.
(TIF)

S5a Fig The maximum acceleration of vertical distribution element.
(TIF)

S5b Fig The RMS of vertical distribution element.
(TIF)

S6a Fig The maximum acceleration of longitudinal distribution element.
(TIF)

S6b Fig The RMS of longitudinal distribution element.
(TIF)

S7 Fig Entropy of Longitudinal distribution element.
(TIF)

S8a Fig Vertically distributed acceleration sensors.
(TIF)

S8b Fig Longitudinal distributed acceleration sensor.
(TIF)

S8c Fig Test instrument.
(TIF)

S8d Fig Structure diagram of distribution acceleration.
(TIF)

S9a Fig The Maximum acceleration of vertical distribution acceleration.
(TIF)

S9b Fig The RMS of vertical distribution acceleration.
(TIF)

S10a Fig Entropy of vertically distributed transverse acceleration.
(TIF)

S10b Fig Entropy of vertically distributed vertical acceleration.
(TIF)

S10c Fig Entropy of vertically distributed longitudinal acceleration.
(TIF)

S11 Fig Comparison of entropy values of vertically distributed acceleration detection points between experimental and simulation results of hammering at position B.
(TIF)

S12a Fig The Maximum acceleration of longitudinal distribution acceleration.
(TIF)

S12b Fig The RMS of longitudinal distribution acceleration.
(TIF)

S13a Fig The RMS of longitudinal distribution acceleration.
(TIF)

S13b Fig Entropy of longitudinal distribution vertical acceleration.
(TIF)

S13c Fig Entropy of longitudinal distribution longitudinal acceleration.
(TIF)

S14 Fig Comparison of entropy values of longitudinal distributed acceleration detection points between experimental and simulation results of hammering at position B.
(TIF)

S1 File Experimental result analysis. (XLS)

S2 File Simulation result analysis. (XLS)
