# Supplementary figures and images for: Study of the spatial distribution of vertical and longitudinal acceleration and sensor installation position of ballast track based on wheel-rail coupling model
Source: PLoS One. 2025 Mar 24;20(3):e0319803. doi: 10.1371/journal.pone.0319803 (PMC11932475; doi:10.1371/journal.pone.0319803)

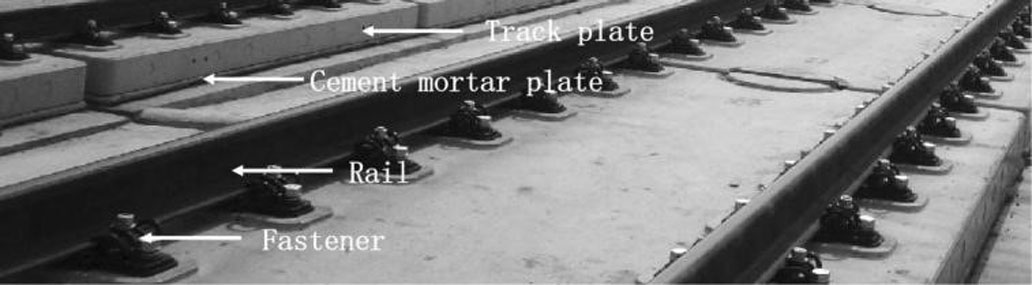

Supplement: S1 Fig — (TIF) [file pone.0319803.s001.tif]

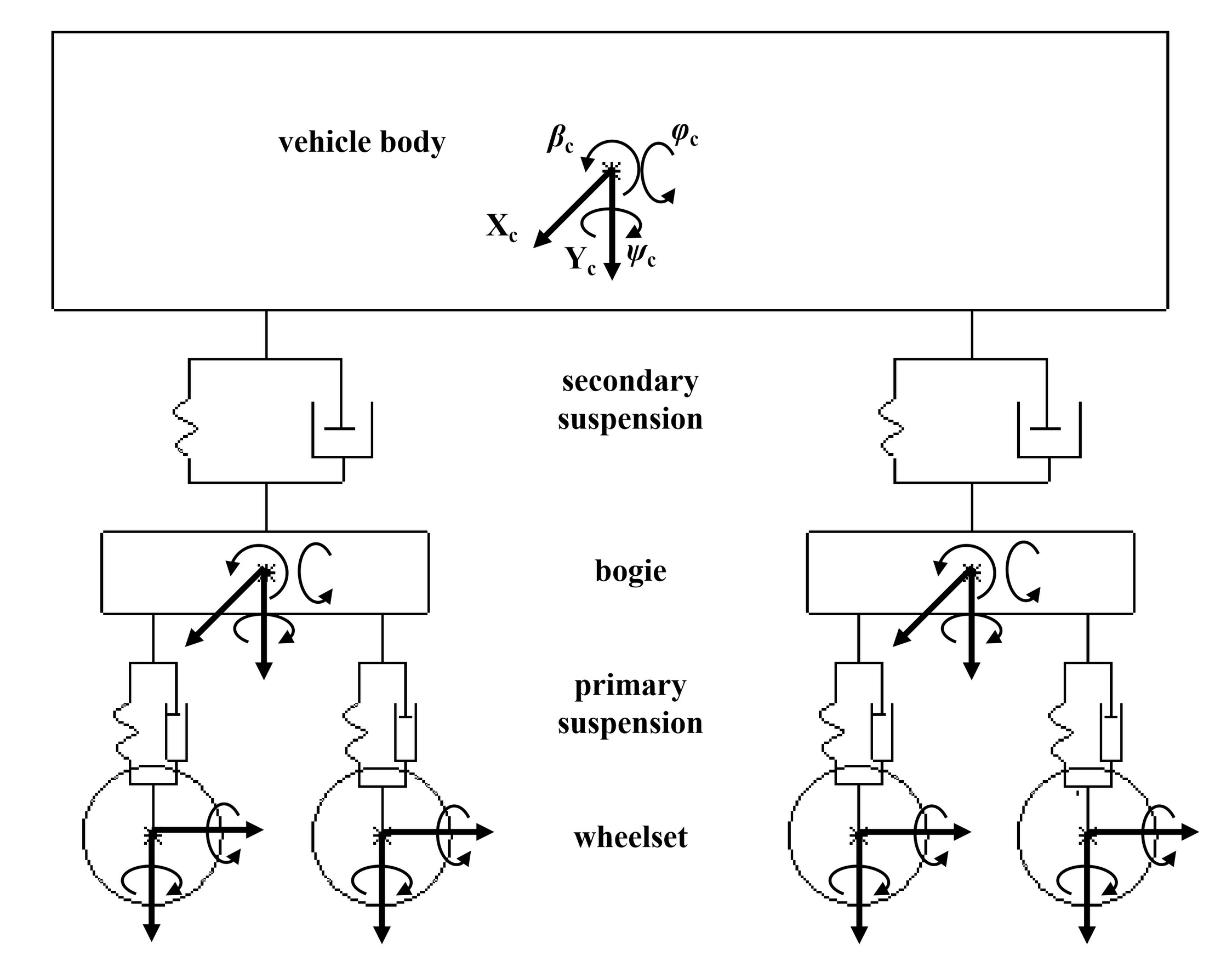

Supplement: S2 Fig — (TIF) [file pone.0319803.s002.tif]

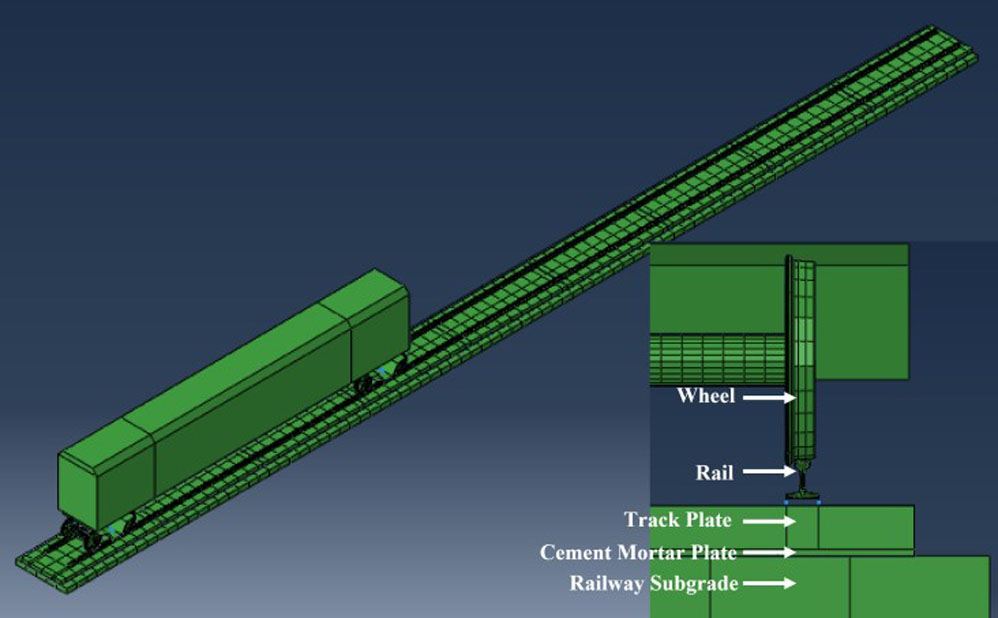

Supplement: S3a Fig — (TIF) [file pone.0319803.s003.tif]

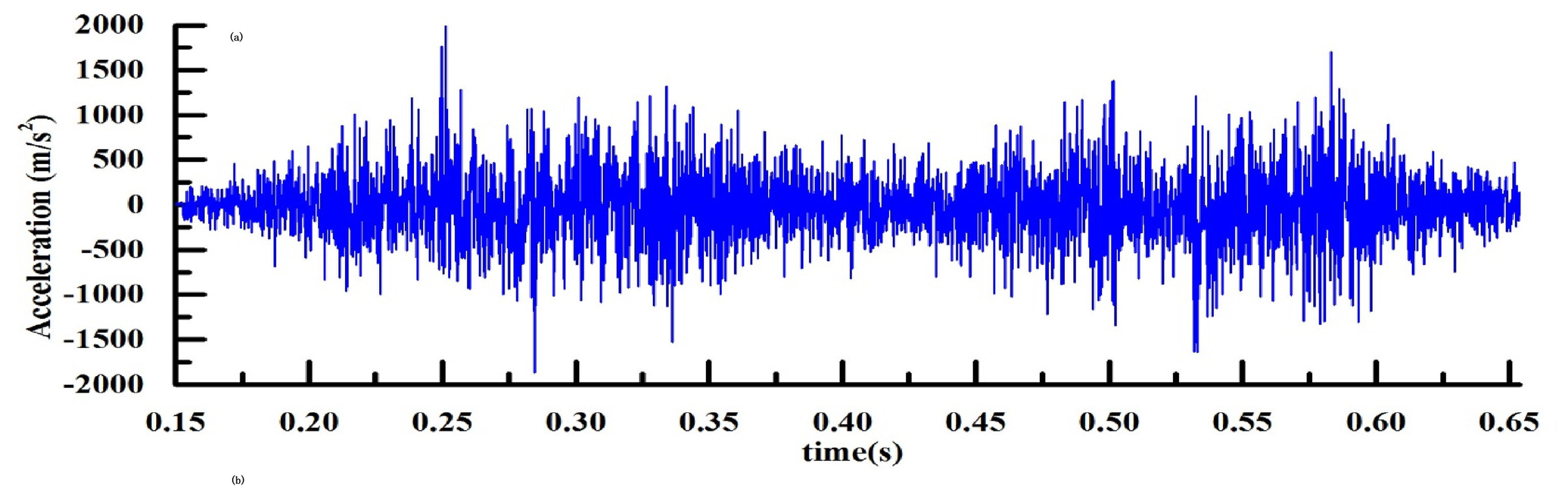

Supplement: S3b Fig — (TIF) [file pone.0319803.s004.tif]

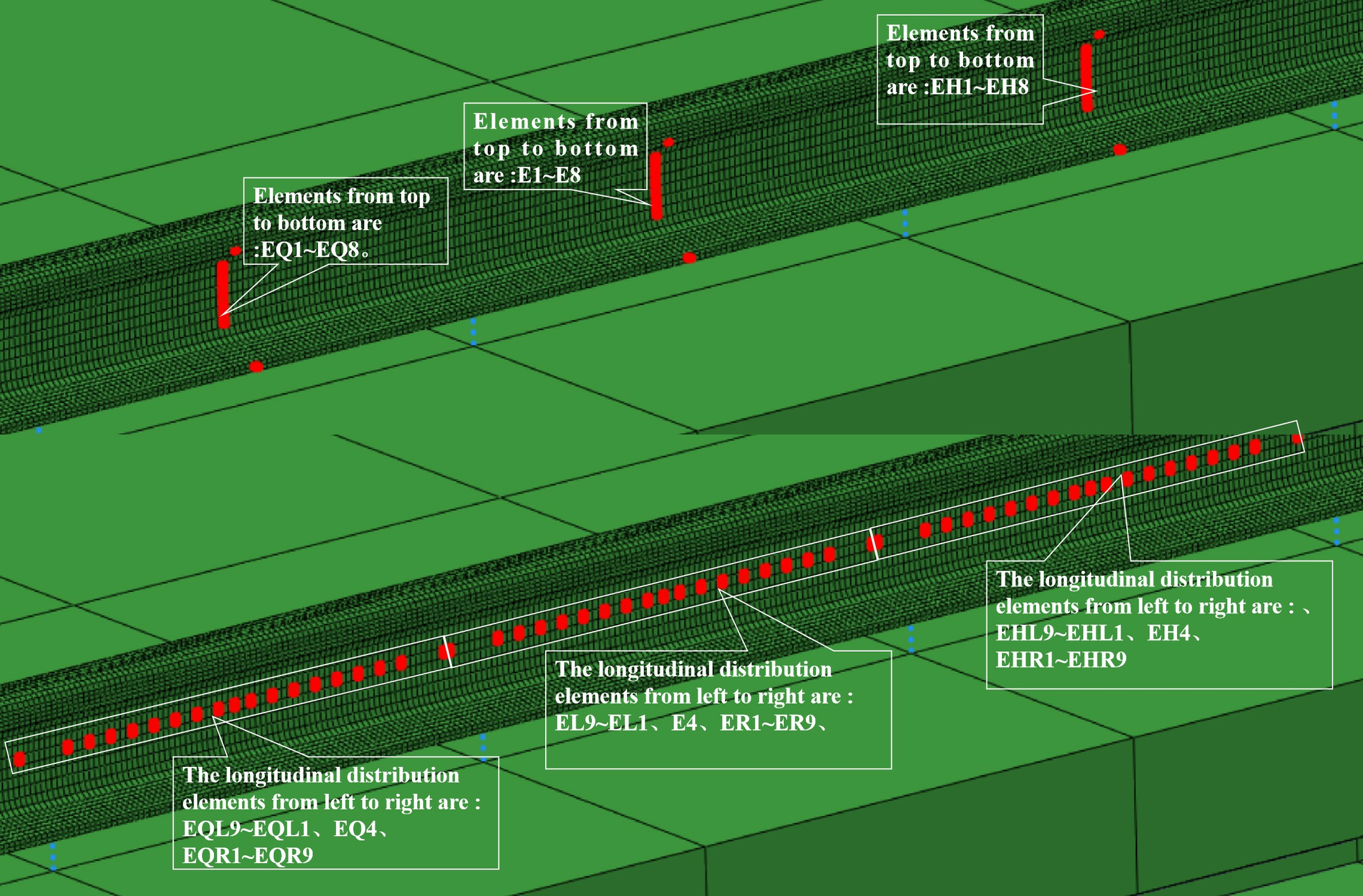

Supplement: S4 Fig — (TIF) [file pone.0319803.s005.tif]

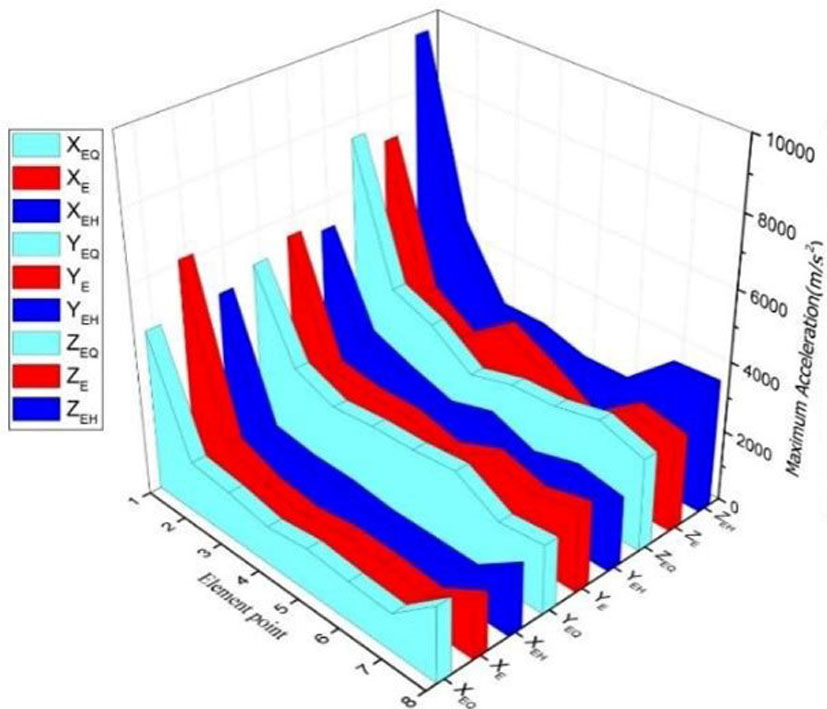

Supplement: S5a Fig — (TIF) [file pone.0319803.s006.tif]

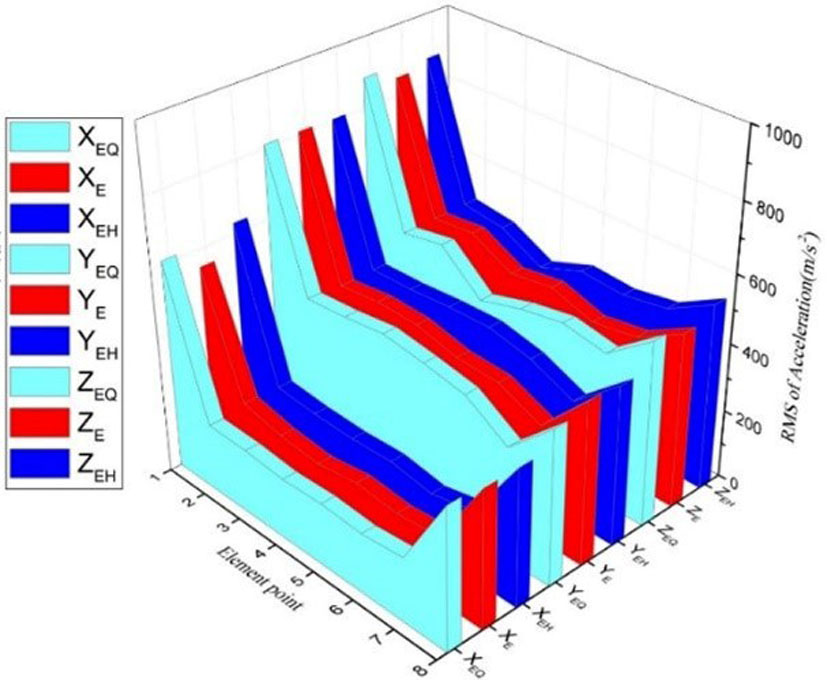

Supplement: S5b Fig — (TIF) [file pone.0319803.s007.tif]

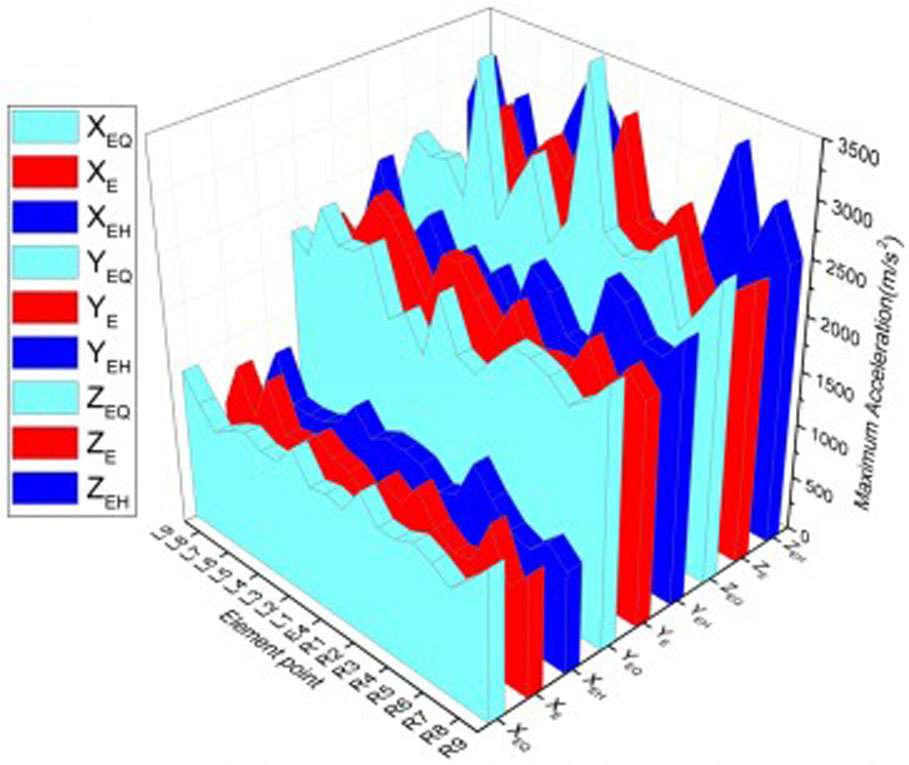

Supplement: S6a Fig — (TIF) [file pone.0319803.s008.tif]

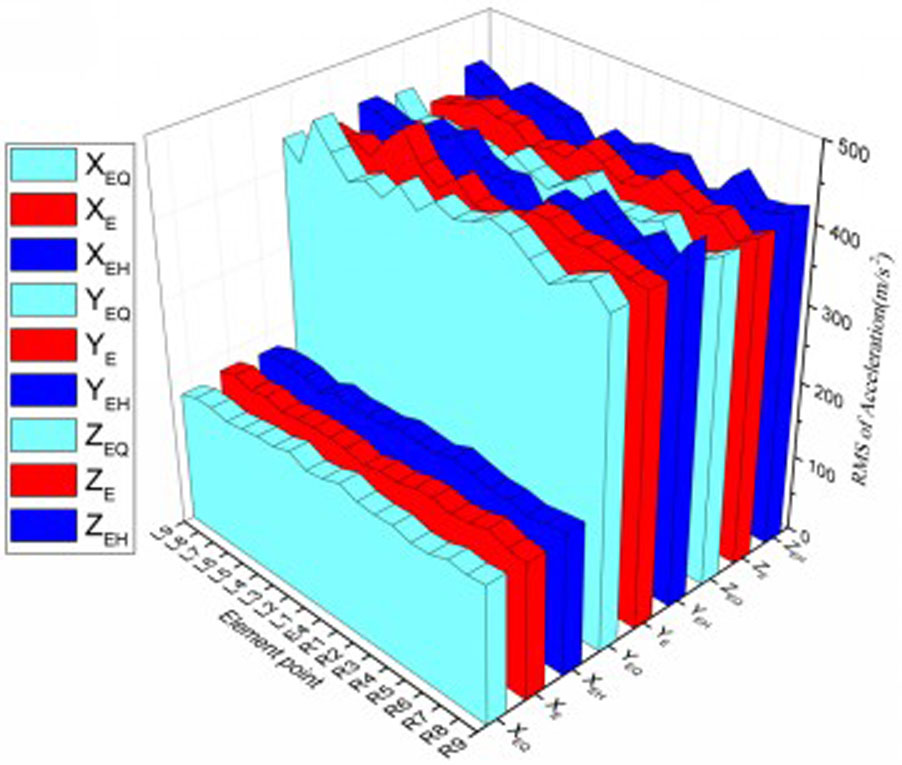

Supplement: S6b Fig — (TIF) [file pone.0319803.s009.tif]

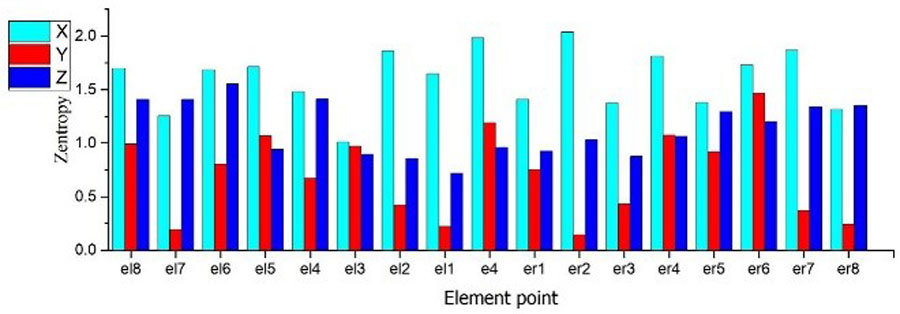

Supplement: S7 Fig — (TIF) [file pone.0319803.s010.tif]

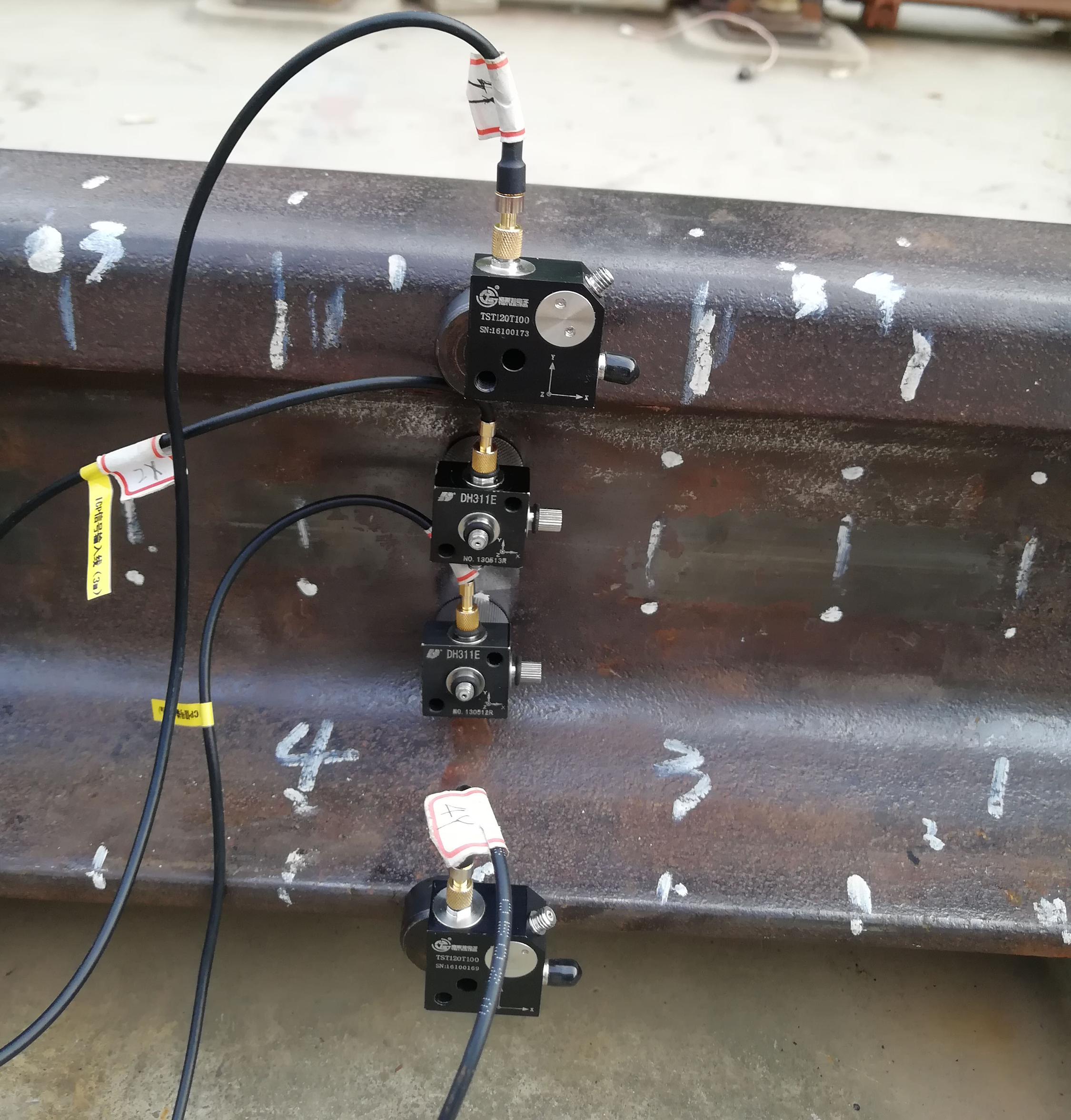

Supplement: S8a Fig — (TIF) [file pone.0319803.s011.tif]

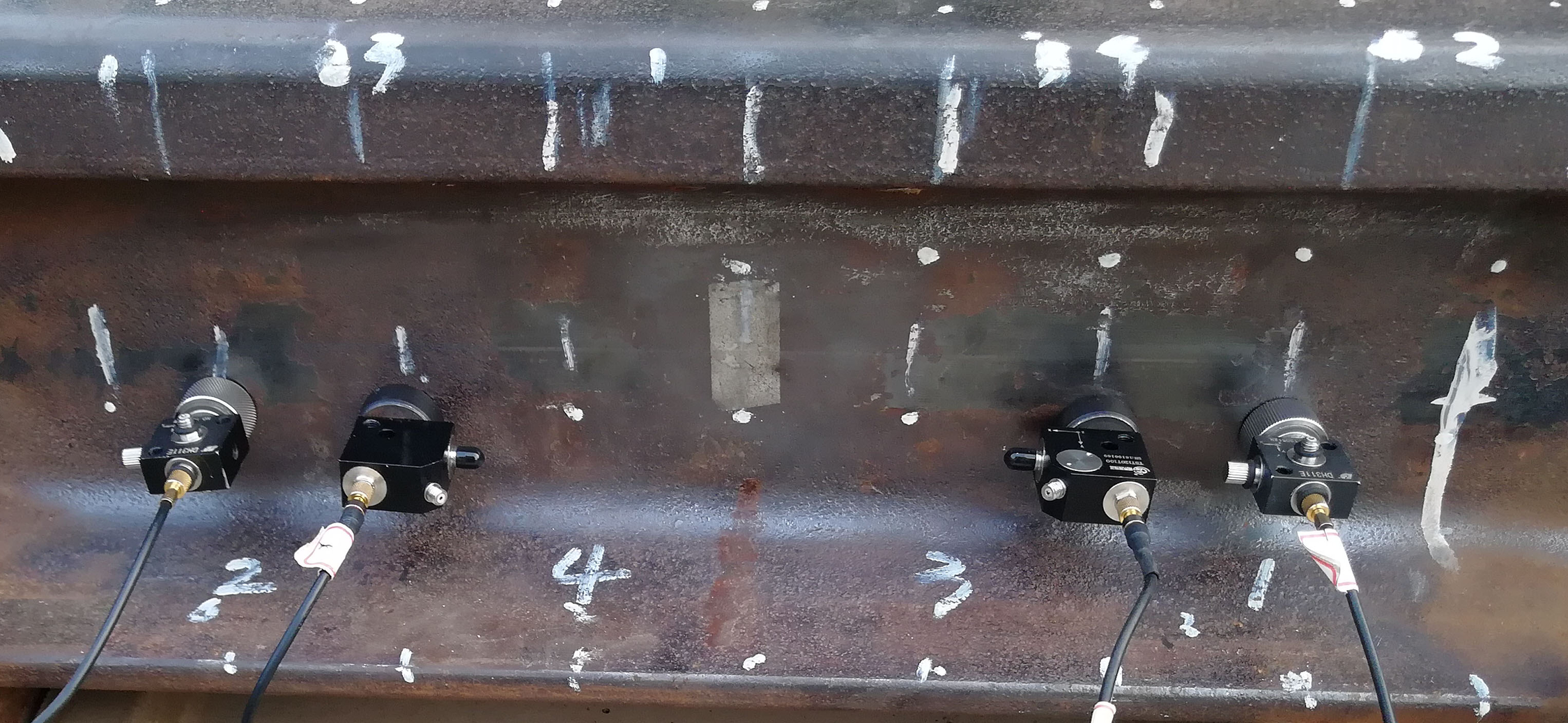

Supplement: S8b Fig — (TIF) [file pone.0319803.s012.tif]

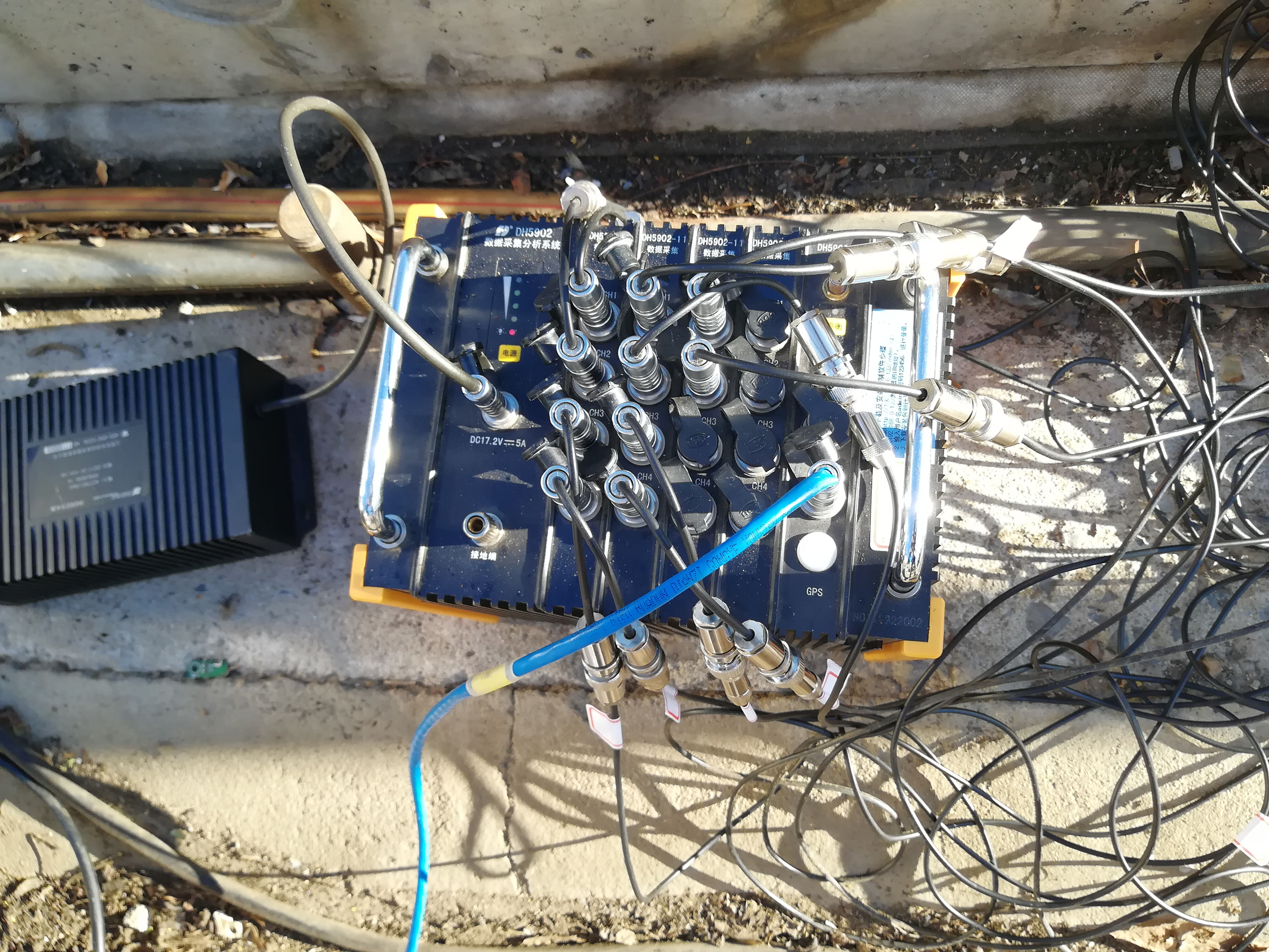

Supplement: S8c Fig — (TIF) [file pone.0319803.s013.tif]

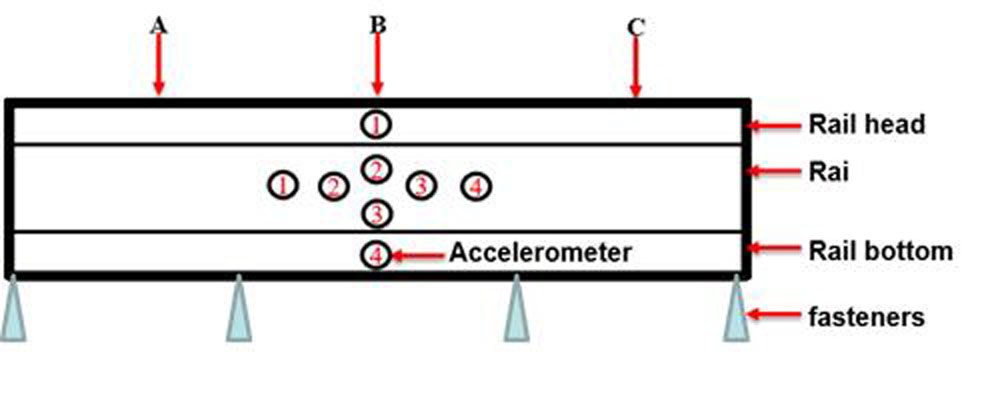

Supplement: S8d Fig — (TIF) [file pone.0319803.s014.tif]

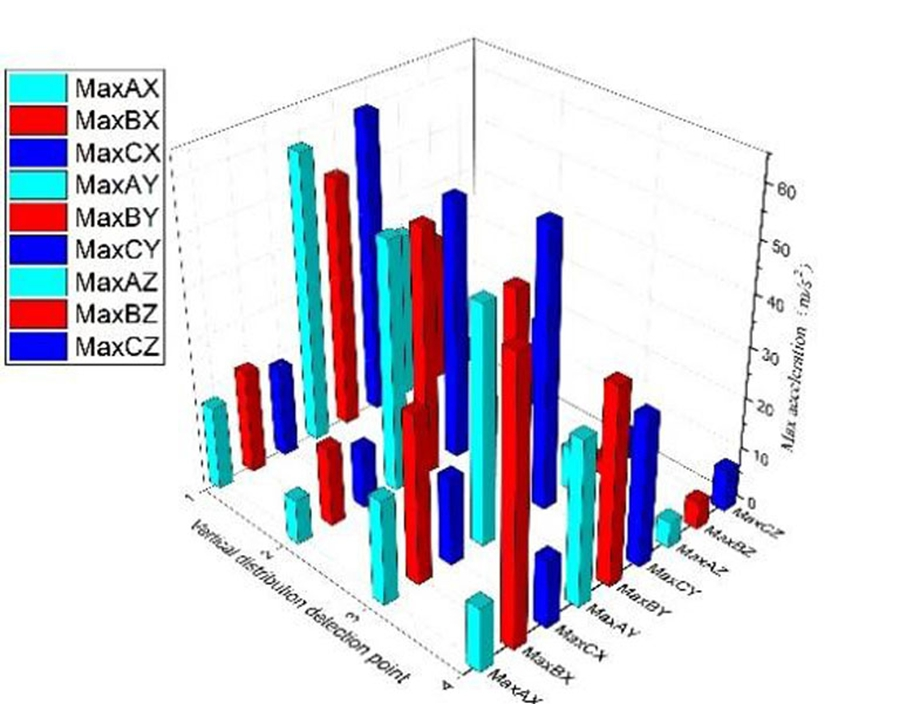

Supplement: S9a Fig — (TIF) [file pone.0319803.s015.tif]

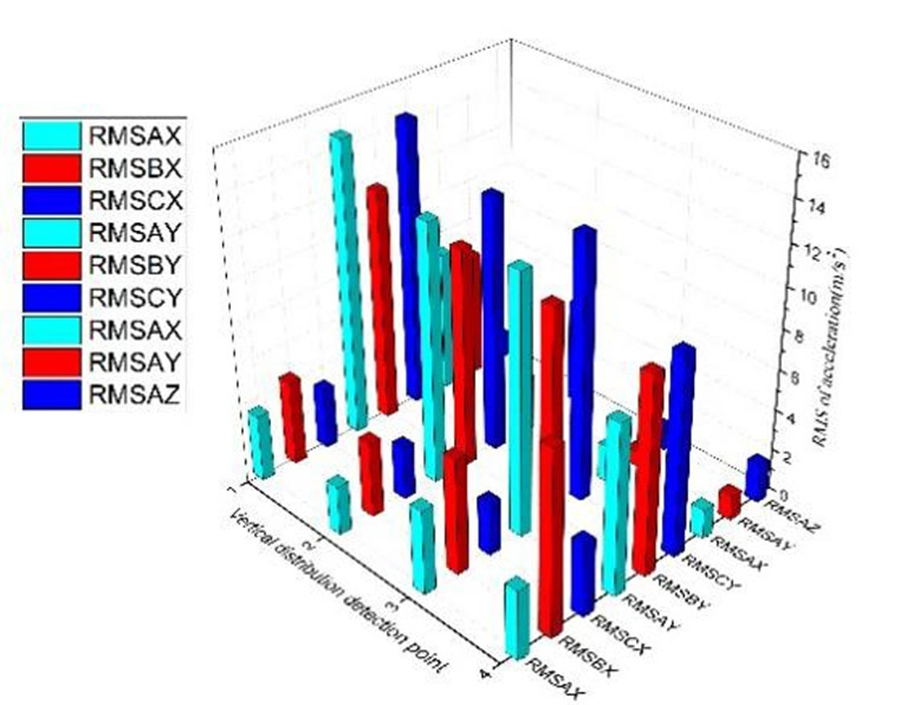

Supplement: S9b Fig — (TIF) [file pone.0319803.s016.tif]

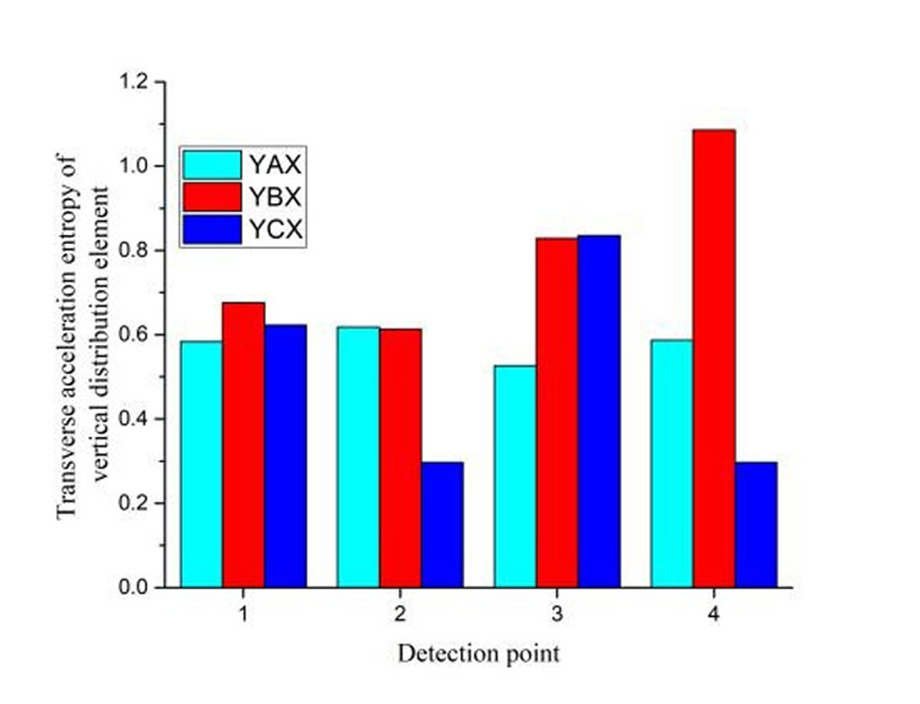

Supplement: S10a Fig — (TIF) [file pone.0319803.s017.tif]

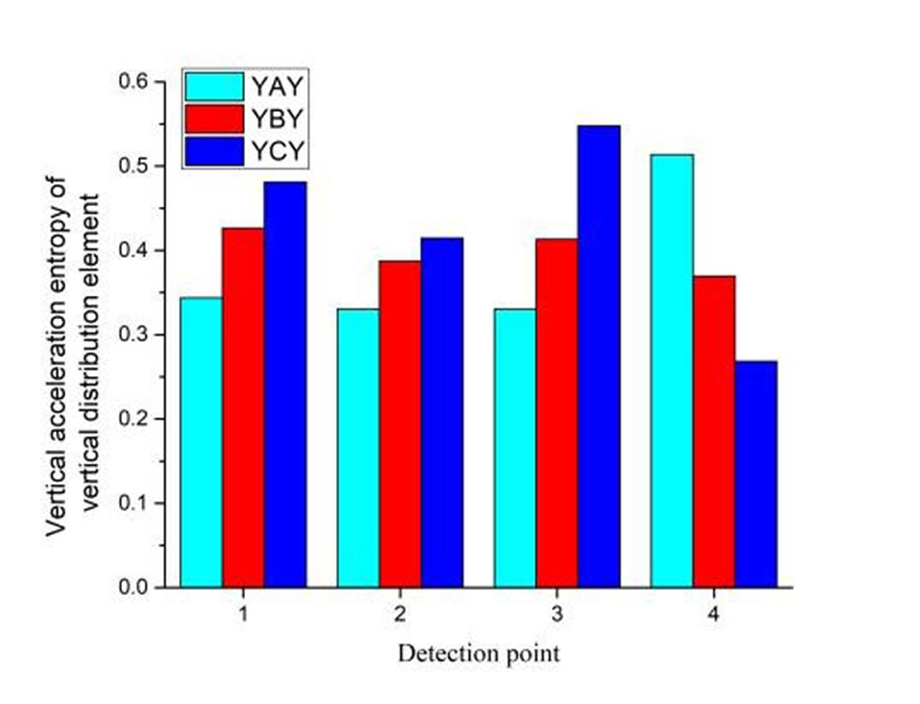

Supplement: S10b Fig — (TIF) [file pone.0319803.s018.tif]

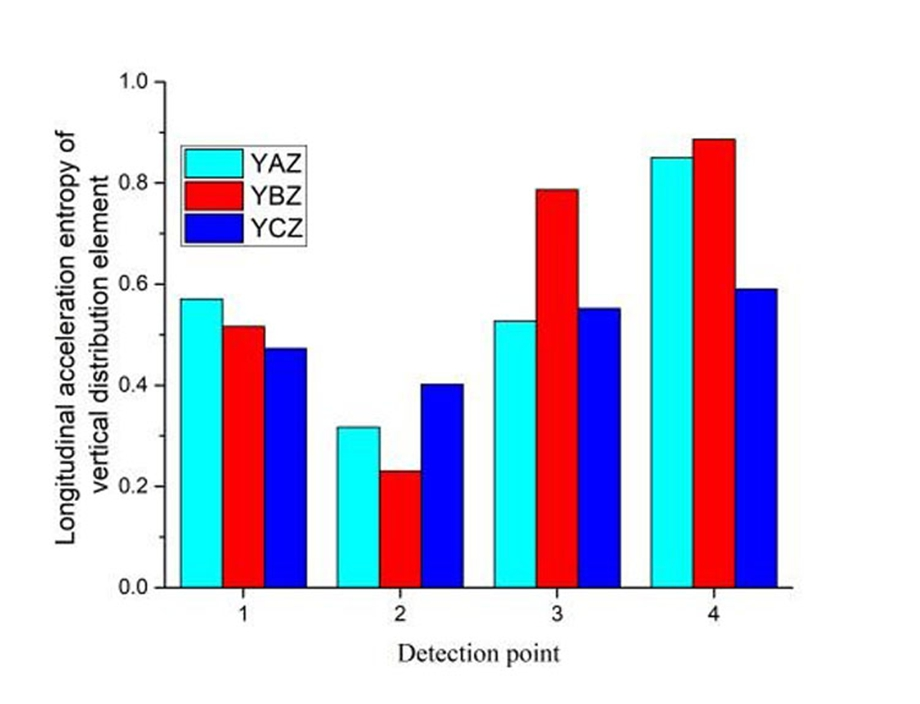

Supplement: S10c Fig — (TIF) [file pone.0319803.s019.tif]

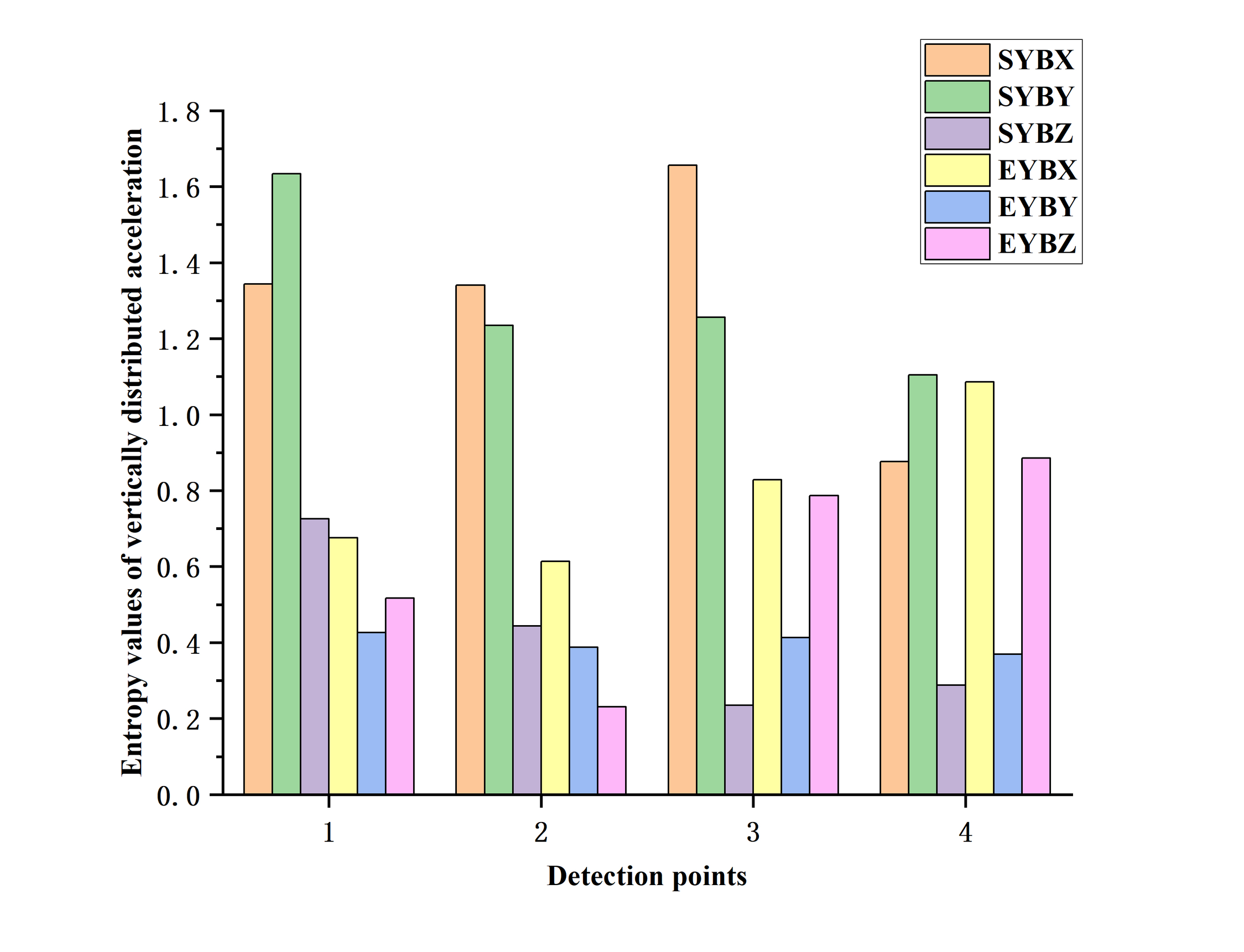

Supplement: S11 Fig — (TIF) [file pone.0319803.s020.tif]

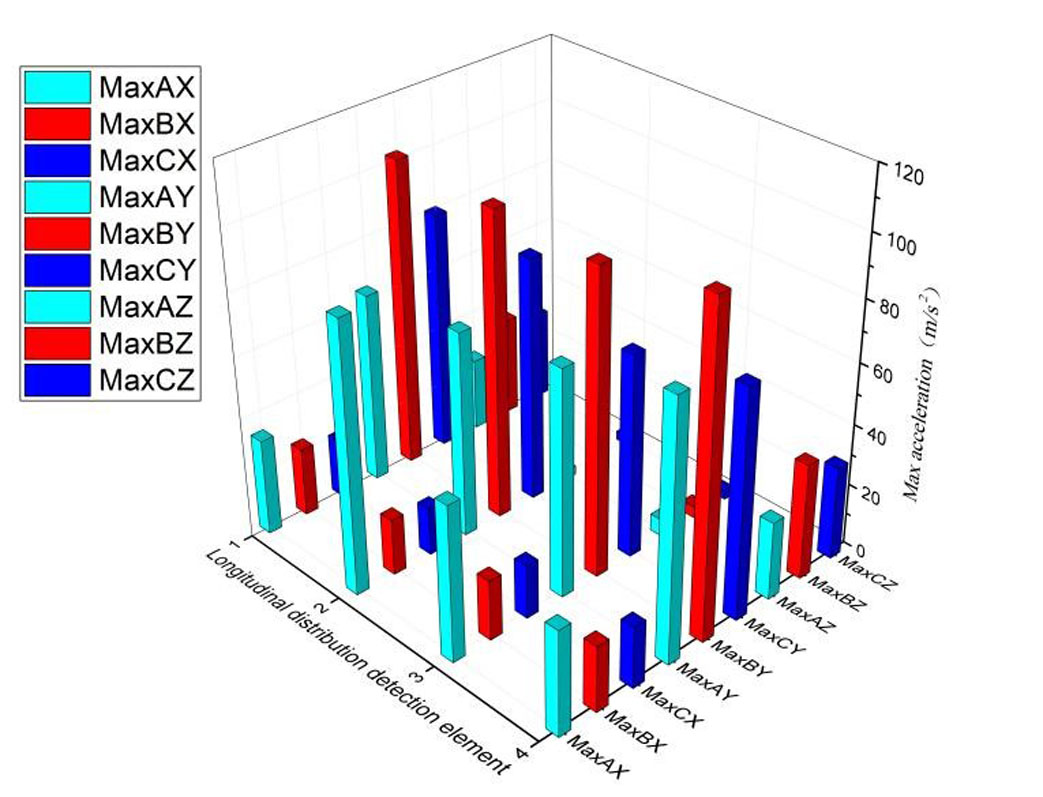

Supplement: S12a Fig — (TIF) [file pone.0319803.s021.tif]

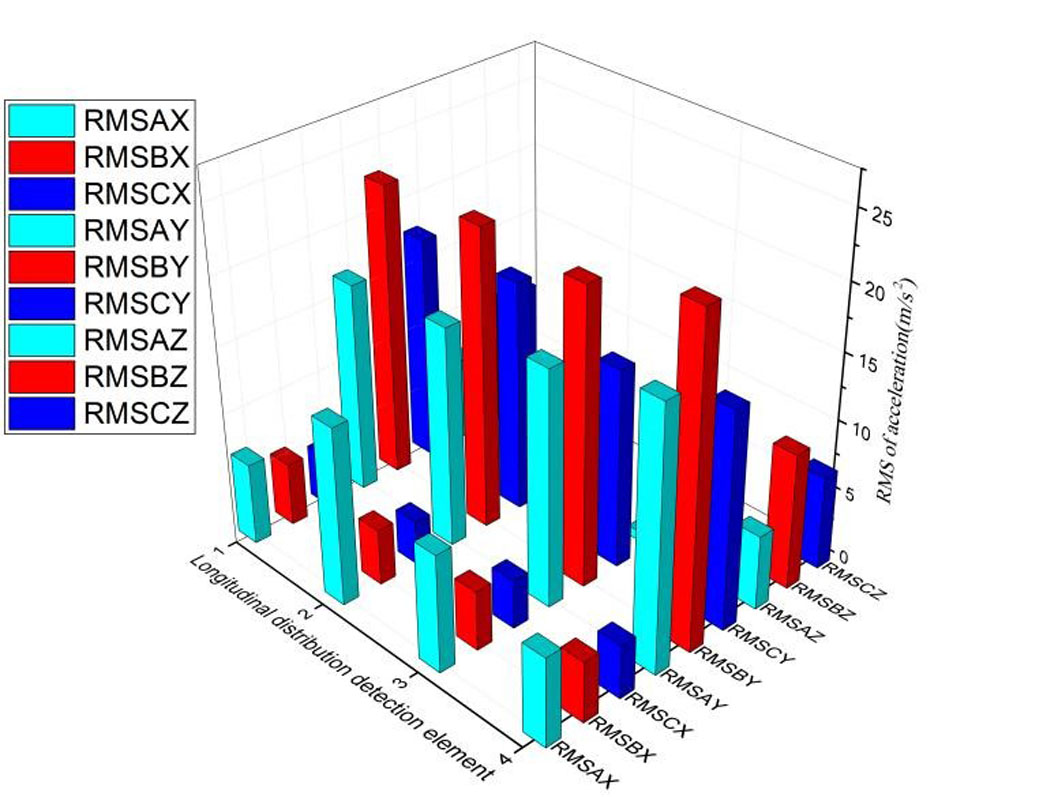

Supplement: S12b Fig — (TIF) [file pone.0319803.s022.tif]

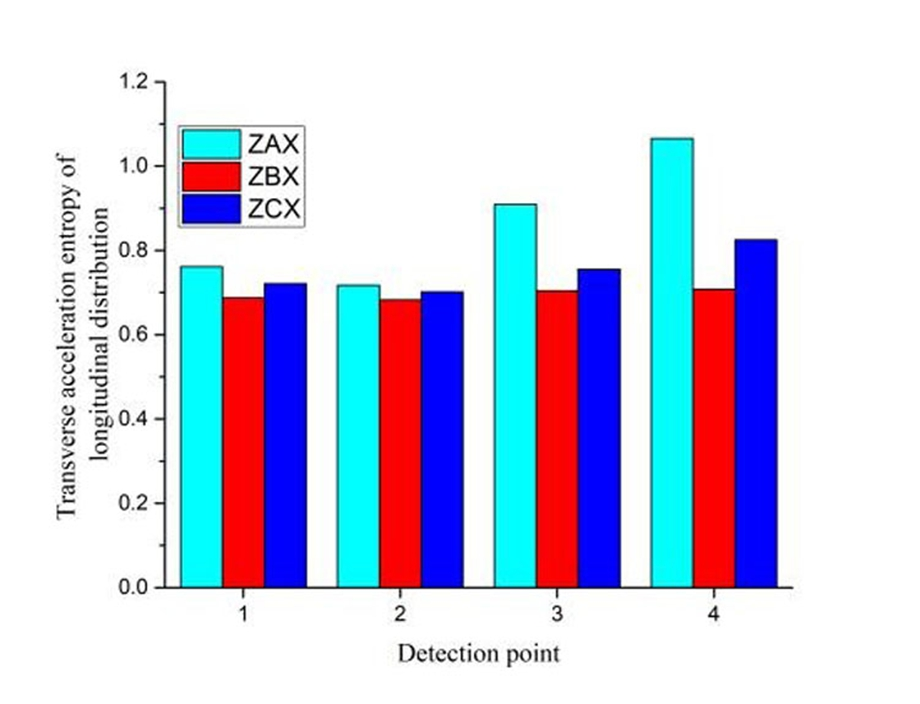

Supplement: S13a Fig — (TIF) [file pone.0319803.s023.tif]

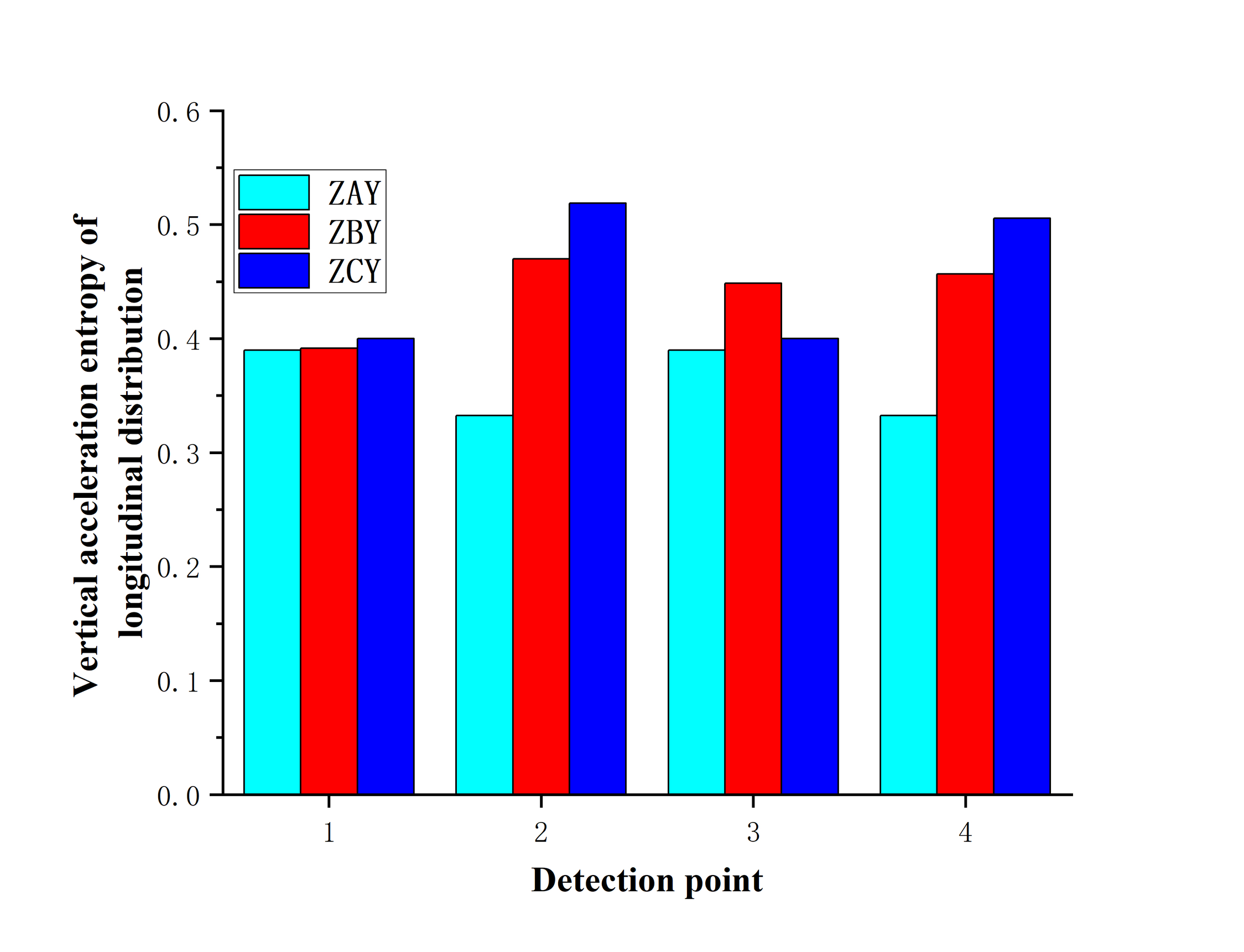

Supplement: S13b Fig — (TIF) [file pone.0319803.s024.tif]

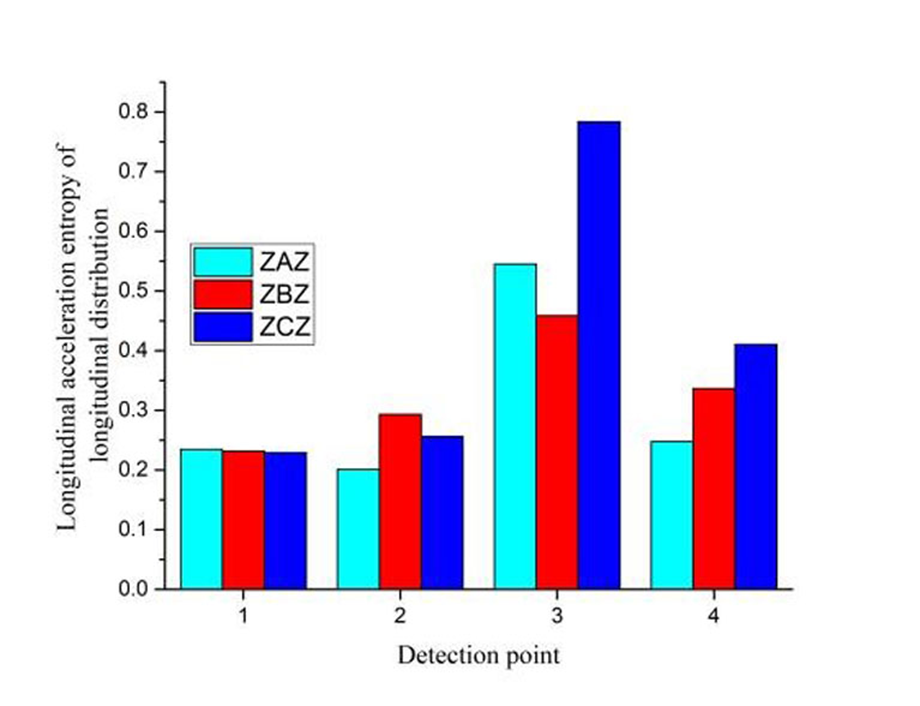

Supplement: S13c Fig — (TIF) [file pone.0319803.s025.tif]

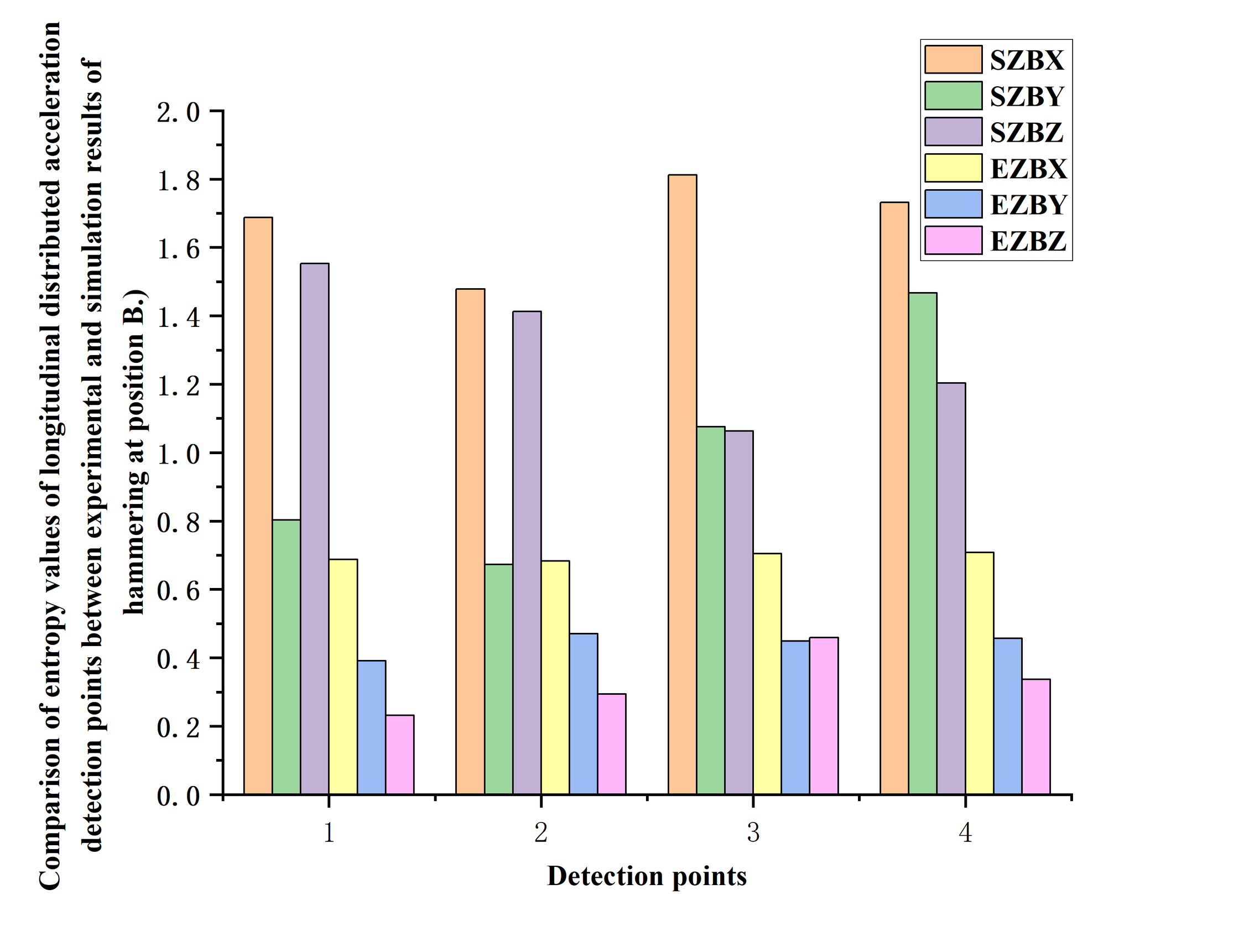

Supplement: S14 Fig — (TIF) [file pone.0319803.s026.tif]
